# A series of replication attempts of experimental awe-induction effects

**DOI:** 10.1371/journal.pone.0356728

**Published:** 2026-09-02

**Authors:** Marianna Graziosi, Alice Chirico, Jeremy D.W. Clifton, Julia S. Rohde, Ria Chhabra, David B. Yaden

**Affiliations:** 1 Johns Hopkins University School of Medicine, Department of Psychiatry and Behavioral Sciences, Baltimore, Maryland, United States of America; 2 Università Cattolica del Sacro Cuore di Milano, Milan, Italy; 3 University of Pennsylvania, Positive Psychology Center, Philadelphia, Pennsylvania, United States of America; 4 Virginia Tech, Department of Clinical Psychology, Blacksburg, Virginia, United States of America; Jinan University, CHINA

## Abstract

The self-transcendent emotion of awe has recently received research attention beyond many other emotions due to several intriguing outcomes reported in the experimental literature. Specifically, after inducing awe, Valdesolo and Graham (2014) found that agency detection is increased, Piff and colleagues (2015) found that prosocial behaviors are more likely, and Rudd and colleagues (2012) found that well-being is enhanced. This article tried to conceptually replicate some of these effects in online and laboratory settings using video, writing, and virtual reality awe inductions. Results from these previous studies were generally not reproduced. To further investigate these failures to reproduce previously reported effects of awe, we conducted three online direct replications with 2.5 times the original sample sizes, and past findings also were not reproduced. We discuss these findings in terms of online research on emotion induction tools and provide recommendations for future research on awe.

## Introduction

Among emotions, and even within the subset of positive emotions, the emotion of awe has a growing field of its own [1]. Awe has been described as a complex emotion due to the mix of valence associated with its experience, which can involve positive feelings such as wonder as well as negative feelings such as fear. After its contemporary introduction as a subject of psychological study by Keltner & Haidt (2003), awe has been studied in correlational and experimental contexts [2]. Some experiments have observed intriguing effects of awe, such as agency detection [3], prosocial behaviors [4], and well-being [5]. We began the present series of studies by attempting to test a mediator of interest by conducting a near replication of a previous study; however, we found that we could not reproduce the main effect. We then conducted a series of conceptual and direct replications of some of the main effects previously reported in the literature on awe.

### Characterizing and measuring awe

The notion of awe can be traced back to the concept of “the sublime” in aesthetics, as articulated by Edmund Burke, Immanuel Kant, and others [6]. Contemporary scholarship has continued to examine the link between awe and the sublime, suggesting that these experiences share core features including perceptual vastness, cognitive accommodation, and complex states of simultaneous positive and negative emotion, including elements of fear [6–8]. Empirical work further supports the importance of subjective and objective features (e.g., height, scale, magnitude) in creating a sense of vastness in both sublime and awe experiences [9,10]. Awe as a distinct psychological construct was first systematically outlined in the seminal paper “Approaching awe, a moral, spiritual, and aesthetic emotion” by Keltner and Haidt. They articulate two necessary qualities of awe: (1) perceptual vastness and (2) a need for accommodation. First, awe involves contact with a perceptually vast stimulus and second, a need for accommodation on behalf of the experiencer, which entails the need for a cognitive shift in one’s understanding of these stimuli [2].

Since Keltner and Haidt’s seminal paper in 2003, researchers have better characterized various facets of awe and developed validated and empirical measures of the awe experience [11,12]. Other facets of awe include alterations to time (e.g., the sense that time slows down or speeds up; [10–12], small self (e.g., the sense that the self is diminished or shrinks), connectedness/oneness (e.g., union or communion with all living things or the universe as a whole), and physiological sensations (e.g., jaw dropping, chills, raised eyebrows). Each of the facets has empirical support, for instance, research has shown that vast objects such as nature [13,14] space [5,15], spiritual [16,17] or psychedelic [18,19] experience or grand theories [20], and *relatively vast* objects such as interpersonally significant experiences with loved ones (e.g., seeing your child walk for the first time; [21]) elicit awe. Research has also demonstrated the need for accommodation, for example awe relates to learning [22,23] and challenges or changes the way that participants viewed the world (e.g., experiencing something that they did not believe was possible; Campos et al., 2013 [24]). Though, it is important to note that operationalizing and eliciting vastness has been much easier for researchers than the assessment and elicitation of a need for accommodation. Awe has also been shown to be associated with alternations to time [15,22,25], a small sense of self [4,17,26,27], feelings of connectedness [26,28], and hallmark physiological sensations such as a dropped jaw, goosebumps, chills, and raised eyebrows [15,29–32]. Each of these refer to the acute subjective effects of awe.

### Outcomes of Awe

The awe literature has produced three findings of particular interest regarding outcomes of experiencing awe. These include increases in: (1) agency detection, (2) prosociality, and (3) life-satisfaction. *Agency detection* refers to “the tendency to interpret events as the consequence of intentional and purpose-driven agents” (Valdesolo & Graham, 2014, pg. 170). *Prosociality* involves “…inclinations to share, care, and assist, which further enable individuals to function more effectively within social collectives” (Piff et al., 2015 pg. 883). *Life-satisfaction*, an overall cognitive assessment of how one’s life is going, is a commonly measured component of subjective well-being (Rudd et al., 2012). Table 1 provides a summary of the original studies on which the work in this paper was based.

**Table 1:** Overview of Original Studies.

Agency Detection – Valdesolo & Graham (2014)		
Study (N)	Awe Induction Method	Outcomes
Study 1* **(N = 120)**	**Videos:** **• awe (panoramic scenes of natural beauty such as mountains)** **• amusement (comedic scenes from nature such as monkeys playing)** **• neutral (an interview)**	**Beliefs in supernatural control increased in the awe condition**
Study 2 (N = 81)	Same videos as Study 1	Uncertainty intolerance mediated the effect of awe on belief in supernatural control
Study 3 (N = 120)	Same videos as Study 1	Agency detection mediates the effect of the manipulation condition on awe
Study 4 (N = 76)	Neutral or Awe Videos from Study 1	Uncertainty intolerance mediated the relationship between awe and agency detection
Study 5 (N = 112)	Videos: • awe – commercial with natural scenes) • amusement – commercial with happy scenes)	awe increased supernatural beliefs
**Prosociality – Piff et al. (2015)**		
Study (N)	Awe Induction Method	Outcomes
Study 1 N = 1519	(no induction)	Dispositional awe is associated with dispositional prosocial tendencies
Study 2* **(N = 75)**	**Writing Recall Task:** **• awe – memory of an awe experience** **• Pride – memory of a pride experience** **• Neutral – memory of a recent event of any kind**	**Ethical decision making increased only in the awe condition**
Study 3 (N = 264)	Videos: • Same as Valdesolo & Graham’s (2014) awe and amusement videos • Neutral – wooden countertop	Increased prosocial values for the awe condition
Study 4	Videos • awe – scientific footage of water droplets colliding at high magnification in slow motion, threatening natural scenes (e.g., tornado) • neutral – wooden countertop	Greater generosity in the dictator game for the awe condition
Study 5	In Vivo • awe – look at tall trees • neutral – look at campus building	Increased helping behavior (picking up pens) and reduced entitlement in the awe condition
**Life Satisfaction – Rudd et al., (2012)**		
Study (N)	Awe Induction Method	Outcomes
Study 1 (N = 63)	Videos • awe – commercial with natural scenes • control – commercial with happy scenes	More perceived time availability in the awe condition
Study 2 (N = 86)	Writing Recall Task • awe – memory of an awe experience • control – memory of a happy experience	Those in awe condition were more willing to donate time and money
Study 3* **(N = 105)**	**Story Visualization** **• awe – read a story about climbing to the top of the Eiffel tower to look out at Paris** **• control – read a story about an unexceptional tower and landscape**	**Those in the awe condition reported more momentary life satisfaction, as well as preference for experiential vs. material goods**

*Note*: Bolded and highlighted denote the specific original studies that conceptual and direct replications were based on.

### The current study

In a series of 6 distinct studies, we attempt to replicate these outcomes associated with awe using two types of replications: conceptual vs. direct [33–35]. The term “conceptual replication” is used throughout the literature to mean repeating studies conceptually, with intentional changes to the methodology to see whether the effects hold under new conditions. On the other hand, a direct replication involves repeating studies conceptually and methodologically as closely as is possible to see if the same result can be reproduced. In the first series of studies in this paper, we conduct conceptual replications, which were designed to expand upon the work of previous authors by including novel hypotheses and multiple outcomes (see Table 1). In the case of direct replications, we consulted with and received materials from the original lead authors to conduct high-fidelity versions of their original work as closely as possible. For all replications the same analytic techniques were used to examine differences between three (ANOVAs) or two (t-tests) conditions, as well as the same partial eta squared (η_p_²) effect size measure to allow for comparison across studies (see Table 2 for an overview of replications).

**Table 2:** Overview of replications.

	N	Stimuli Format	Stimuli Content	Outcome of Interest
**Conceptual Replication** **Studies**				
*Study 1*	625	Video	Panoramic scenes of nature	Agency detection, Prosociality, & Well-Being
*Study 2*	570	Writing Recollection Task	Write about a memory of an awe experience	Agency detection, Prosociality, & Well-Being
*Study 3*	91	Virtual Reality Task	Experience of zooming out and viewing earth from space	Agency detection, Prosociality, & Well-Being
**Direct Replication** **Studies**				
*Study 4* (Study 1 from Valdesolo & Graham, 2014)	300	Video	Panoramic scenes of nature	Supernatural Belief Belief in God
*Study 5* (Study 2 from Piff et al, 2015)	188	Writing Recollection Task	Write about a memory of an awe experience	Small Self Ethical Decision Making
*Study 6* (Study 3 from Rudd et al., 2012)	263	Reading Task	Read about climbing Eiffel tower and looking out at Paris	Momentary Life Satisfaction Preference for Experiential v. Material Goods

Data collection for these studies took place between January 2018 and January 2019. Study 1 uses video manipulations to test all three outcomes (agency detection, prosocial behavior and life satisfaction). Study 2 follows the same methodology, using writing and recall manipulations, and finally Study 3 utilizes virtual reality to test the associations between awe and these three outcomes. Following (unexpected) replication failures in these conceptual replications, we conducted a second series of three studies which were direct replications of the three studies mentioned in the introduction, but with larger sample sizes. Data collection for these studies took place between February 2025 and April 2025. Study 4 is a direct replication of Study 1 from Valdesolo & Graham (2014) looking at awe and agency detection, Study 5 is a direct replication of Study 2 from Piff et al.‘s (2015) study examining awe and prosocial behavior, and Study 6 is a direct replication of Study 3 from Rudd et al.’s (2012) paper on awe and life satisfaction. In the subsequent sections, first the conceptual replications are described, followed by the direct replications.

### Conceptual replications

In this present series of studies, we attempted to replicate these three findings—that awe increases (1) agency detection, (2) prosociality, and (3) well-being—and sought to investigate whether these three outcomes bore a relation to each other. Specifically, our initial hypothesis was that agency detection would mediate the relationship between the experience of awe and the outcomes of prosociality and well-being. Additionally, we were interested in the exploratory question of whether different aspects of awe in the awe experience scale (AWE-S; 9) – such as perception of vastness, need for accommodation, altered perception of time, self-diminishment, connectedness, or physiological reactions––related differentially to different outcomes.

We could not explore these hypotheses because foundational findings from the literature did not replicate under the current conditions in online and laboratory-based conceptual replication attempts of the bolded studies described in the previous section [3–5].The previous research reviewed above included, on average, 107 participants per study (ranging from 63 to 264), whereas the present research (except Study 3) includes anywhere from twice to ten times the number of participants (average of 598). We therefore exceed the 2.5 times sample size rule of thumb described by Simonsohn (2015) [36]. In most cases in the present studies, the emotion induction techniques and outcome measures are identical to those used in the previous research. Because the first two studies rely on online participants recruited via mTurk, Study 3 utilized a more immersive, laboratory-based awe induction technique to replicate findings.

It should be noted that although data for two of the six studies were collected via Amazon Mechanical Turk, concerns regarding participant non-naivety and response trustworthiness were addressed through multiple quality assurance procedures (e.g., attention checks, eligibility screening, and exclusion of low-quality responses), although these concerns remain an inherent limitation of online convenience samples. All data have been made publicly available on the Open Science Framework repository and can be accessed using the following identifier: DOI 10.17605/OSF.IO/VD26U. We report how we determined our sample size (above; Table 1), all data exclusions (if any), all manipulations, and all measures in the study.

### Study 1: Video Manipulations

Study 1 study used videos to elicit awe, amusement, or no emotion (neutral). A battery of questionnaires was then administered. We expected the experimental awe condition would result in changes to supernatural beliefs, agency detection, prosocial beliefs, and well-being relative to the active and passive control conditions.

### Participants

A total of 755 Participants were recruited using the online survey platform Amazon Mechanical Turk (M-Turk). Participants provided written informed consent and agreed that they were over 18 years of age. Upon completion of the survey, participants were compensated two dollars for their participation in the study. This study was approved by the first author’s Institutional Review Board at the time the conceptual replications were conducted (Long Island University, protocol #18/01–001).

Of the initial participants, 130 were removed from the study: 1 for failing to give consent to participate; 65 for answering “yes” when asked whether they had seen the video before; 5 for failing the attention check question of “Please write something you remember from the video” (if they didn’t respond or responded with an irrelevant answer). Lastly, 59 participants were removed for submitting an incomplete survey.

**Participant Characteristics.** In the Awe condition (*N =* 220), the mean age was 36.89 (*SD =* 10.95), 51.8% male, and 79.5% non-Hispanic white, 10.9% Black, and 5.9% Asian or Asian America. In the amusement condition (*N =* 178), the mean age was 35.83 (*SD =* 10.17), 55.1% male, and 77.0% non-Hispanic white, 8.4% black, and 7.9% Asian or Asian American. Finally, in the Neutral condition (*N =* 227), the mean age was 36.75 (*SD =* 10.70), 55.9% male, and 84.1% non-Hispanic white, 6.2% black, and 5.3% Asian or Asian American.

## Materials

**Emotion Induction Videos.** Videos to induce feelings of awe (experimental), amusement (control), or a neutral (control) emotion were selected based on videos used in past research [3–5]. The participants in the awe condition watched a video of mountains and sweeping views of nature which was roughly four minutes long (3:57) and included time-lapse footage of perceptually vast footage of Yosemite National Park. The participants in the amusement condition watched a video of comedic footage of animals in nature (4:34). The participants in the neutral condition watched a video of an interview conducted in 1957 about architecture (1:47).

### Measures

Internal reliabilities for all measures are available in the supplement (S1 Table). In Study 1, all scales and subscales were reliable (*α* > .70), except the *Baby Scale* of the *Mind Perception Scale* (*α* = .57).

**Positive and Negative Affect.** Items from the Positive and Negative Affect Scale developed by [37] measured both positive and negative emotion. For Negative Affect, participants were presented with statements such as “In this moment I feel anxious,” and then asked to evaluate the degree to which they felt the given emotion on a likert-type scale from ranging from 1 “Least intensely” −11 “Most intensely”. For positive Affect, participants were presented with statements such as “In this moment I feel happy,” and then asked to evaluate the degree to which they felt the given emotion. A single-item measure of awe was embedded into the PANAS following the same format: “In this moment I feel awe”.

**Awe.** The Awe Experience Scale (AWE-S; Yaden et al., 2019) measures various qualities associated with awe experiences and contains six subscales: vastness, accommodation, self-diminishment, connectedness, time alteration, and physiological reactions. Participants evaluated the degree to which they agreed with statements such as “I felt my sense of self shrink” from 1 “Strongly Disagree” to 7 “Strongly Agree”.

**Intentional Pattern Perception.** Intentional pattern perception was measured using a numerical judgment task adopted from past research by Valdesolo and Graham (2014). In this task which consisted of 10 trials, participants are asked to distinguish between random strings of numbers that are either computer generated, or human produced.

**Supernatural Belief.** Supernatural Belief was measured using questions adopted from past research by Valdesolo and Graham (2014). Given that supernatural control (i.e., the idea that the universe is controlled by God or a supernatural force) was the measure of interest in past research by Valdesolo and Graham (2014), the three-item index of belief in supernatural control [38] was also scored separately. These items were assessed on a 10-item likert-type scale.

**Need for Cognitive Closure.** Given that Valdesolo and Graham (2014) used the construct of Cognitive Closure to measure uncertainty tolerance, the Need for Closure-Short Form Scale developed by [39] was used in this study to measure uncertainty tolerance on a likert-type scale ranging from 1 “Strongly Disagree” −6 “Strongly Agree”. Valdesolo and Graham (2014) focused on the ambiguity subscale of the Need for Closure scale in their past work. For this reason, intolerance of ambiguity was scored separately.

**Mind Perception.** Gray et al.‘s (2007) Mind Perception Scale [40] was used to measure mind perception in this study. The scale contains 48 questions, with 6 questions organized around a given target (e.g., adult human, baby, dead woman, dog, God, robot, superman, and tree). Each of the items is evaluated on a likert-type scale ranging from 0 “Not at all” to 6 “Very much”.

**Prosocial Tendencies.** Given that Piff and colleagues (2015) assessed awe and its relationship to prosocial behavior, Carlo et al.‘s (2003) Prosocial Tendencies Measure [41] was used to measure prosocial behavior in this study. Items such as “I can help others best when people are watching me” were evaluated on a likert-type scale from 1 “Describes me not at all” to 5 “Describes me greatly”.

**Life Satisfaction.** Given that Rudd, Vohs, and Aker (2012) used The Satisfaction with Life Scale (SWLS; Diener et al., 1985) [42] this scale was used to measure life satisfaction using a 7-item likert-type scale ranging from 1 “Strongly disagree” to 7 “Strongly Agree”.

### Procedure

The survey was created using Qualtrics survey software and distributed on M-Turk, an online survey platform. Once they had given their consent to participate and filled out demographic information, participants were randomly assigned to one of the three video conditions: the awe video (experimental), the amusement video (control), or the neutral affect video (control). After watching the video, the rest of the survey was the same for all participant and all participants received the survey in the same order.

## Results

**Fig 1:**
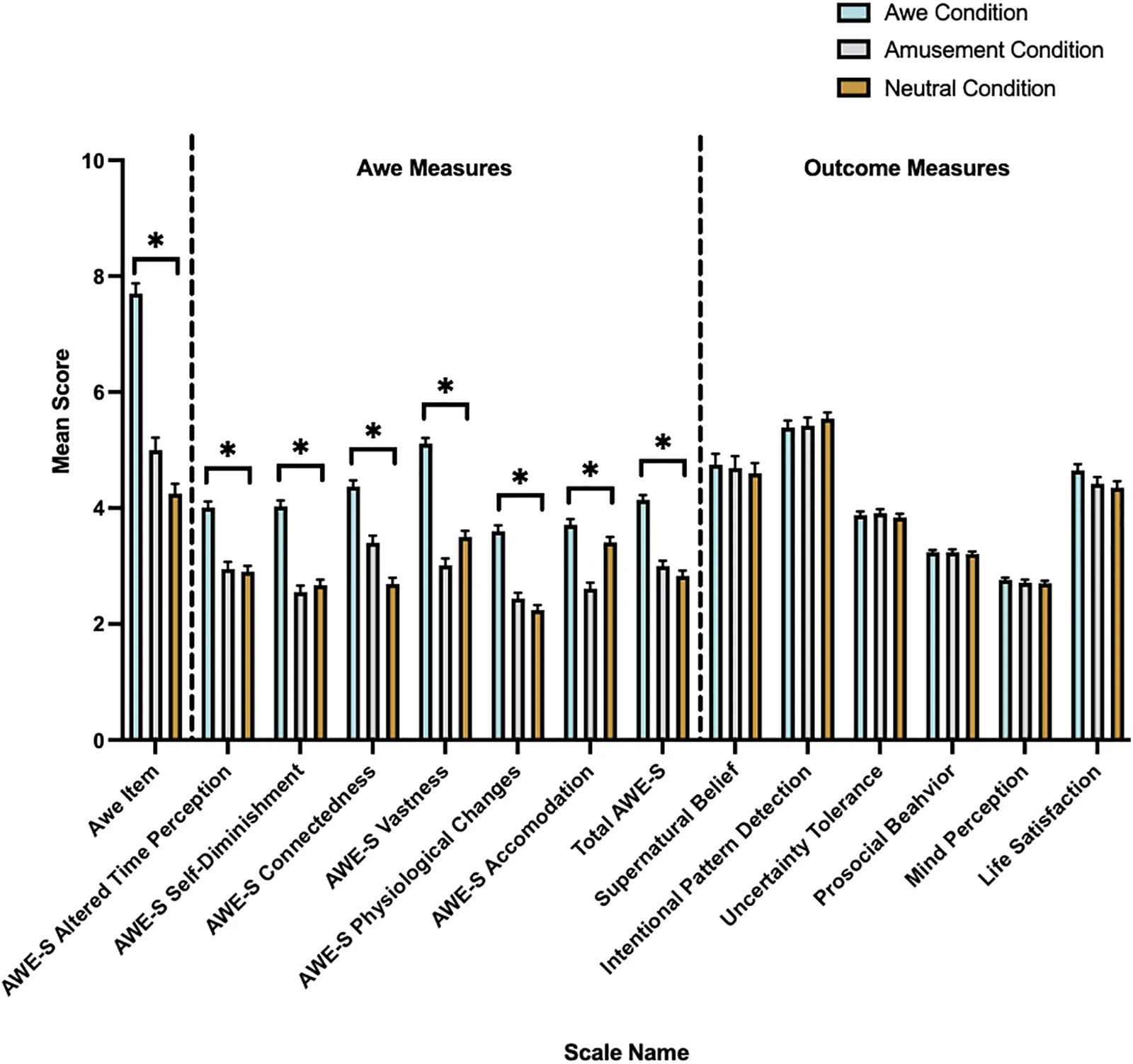
Mean Scores of Awe and Outcome Measures for Study 1. Note: Awe Condition *n =* 220; Amusement Condition *n =* 178; Neutral Condition *n* = 227. The Awe Item was used as manipulation check. An asterisk denotes a statistically significant difference at the p < .01 level of an omnibus effect between all three conditions. Error bars represent standard error.

**Manipulation Checks.** Planned Contrasts revealed that participants reported feeling more awe on an awe item embedded into the PANAS scale in the awe condition (*M* = 7.70, *SD* = 2.57) than the amusement (*M* = 5.0, *SD* = 2.87) or neutral (*M* = 4.25, *SD* = 2.56) conditions, [*F*(2, 624) = 102.630, *p* < .001, *η*_*p*_*²* = .25] (see Fig 1).

More negative affect was self-reported in the neutral condition (*M* = 2.99, *SD* = 1.74) than in the amusement (*M* = 2.51, *SD* = 1.77) or awe (*M* = 2.21, *SD* = 1.40) conditions, [*F*(2,624) = 12.806, *p* < .001, *η*_*p*_*²* = .04]. While more positive affect (calculated *without* the embedded awe item) was reported for the amusement condition (*M* = 6.22, *SD* = 2.34) than the neutral condition (*M* = 5.13, *SD* = 2.05), scores on positive affect were significantly higher in the awe (*M* = 6.98, *SD* = 1.93) condition compared to either the amusement or neutral conditions, [*F*(2, 624) = 44.221, *p* < .001, *η*_*p*_*²* = .06]. Table 3 provides an overview of the effect sizes of the awe manipulations in the original and replication studies.

**Table 3:** Awe Manipulation Effect Size Comparison to Original Studies.

*Original Study*	*Replication*
*Valdesolo & Graham (2014)* *• Study 1 –* ηp² *= .47* (large)	*Conceptual* *• Study 1 –* ηp² *= .25* (large) *Direct* *• Study 4 –* ηp² *= .30* (large)
*Piff et al. (2015)* *• Study 2 – NR*	*Conceptual* *• Study 2 –* ηp² *= .15* (large) *Direct* *• Study 5 –* ηp² *= .31* (large)
*Rudd et al. (2012)* *• Study 3 –* ηp² *= .04* (small)	*Conceptual* *• Study 3 –* ηp² *= .09* (medium) *Direct* *• Study 6 – η*_*p*_*² = .01*^*NS*^ (small)

*Note*. NR = Not reported. NS = Nonsignificant effect. Effect magnitude labels correspond to conventional benchmarks for partial eta squared (*ηp²*): small = .01, medium = .06, large = .14 [43,44],

**Outcome measures.** Planned Contrasts revealed that participants scored significantly higher on awe as measured by the AWE-S total (see Table 4) in the awe condition (*M* = 4.14, *SD* = 1.26) than in the neutral (*M* = 3.00, *SD* = 1.23) or amusement (*M* = 2.83, *SD* = 1.35) conditions, [*F* =(2, 624) = 65.245, *p* < .001, *η*_*p*_*²* = .17]. The awe item in the PANAS scale and the AWE-S scale were found to be significantly correlated, *r* = .67, *p < .*001. Furthermore, participants scored significantly higher on each dimension of awe measured by the AWE-S (i.e., altered perception of time, connectedness, self-diminishment, vastness, physiological changes, accommodation, and connectedness) in the awe condition than in either the amusement or neutral conditions. There were no significant differences in outcomes across conditions (See Table 5; more detail provided in S1 File). Although the experimental manipulations generally did not produce significant between-group differences, exploratory analyses indicated that participants reporting greater subjective experiences of awe tended to report higher scores on several outcome measures (see S3 Table for exploratory correlations for each of the six studies in this paper).

**Table 4:** Group differences in factors of the awe experience scale in study 1.

Dimension of Awe	Awe Condition (N = 220)	Amusement Condition (N = 178)	Neutral Condition (N = 227)	*F*	*p*	*η*_*p*_ *²*
Altered Perception of Time	M = 4.01, SD = 1.54	M = 2.95, SD = 1.61	M = 2.90, SD = 1.54	35.029	<.001*	.10
Self-Diminishment	M = 4.03, SD = 1.53	M = 2.55, SD = 1.50	M = 2.68, SD = 1.41	64.153	<.001*	.17
Connectedness	M = 4.37, SD = 1.60	M = 3.40, SD = 1.65	M = 3.23 SD = 1.61	31.116	<.001*	.09
Vastness	M = 5.11, SD = 1.47	M = 3.01, SD = 1.62	M = 3.50, SD = 1.62	100.804	<.001*	.25
Physiological Changes	M = 3.60, SD = 1.51	M = 2.44, SD = 1.34	M = 2.24, SD = 1.30	60.674	<.001*	.16
Accommodation	M = 3.71 SD = 1.49	M = 2.61, SD = 1.36	M = 3.41, SD = 1.38	31.486	<.001*	.09
Total AWE-S	M = 4.14 SD = 1.26	M = 3.00 SD = 1.23	M = 2.83 SD = 1.35	65.245	<.001*	.17

**Table 5:** Group differences in outcomes in study 1.

Outcome	Awe Condition (N = 220)	Amusement Condition (N = 178)	Neutral Condition (N = 227)	*F*	*p*	*η*_*p*_ *²*
Supernatural Belief	M = 4.75, SD = 2.74	M = 4.69, SD = 2.77	M = 4.60 SD = 2.69	.161	.851	.00
Intentional Pattern Perception	M = 5.39, SD = 1.77	M = 5.42, SD = 1.85	M = 5.54, SD = 1.61	.468	.626	.00
Uncertainty Tolerance	M = 3.88, SD = 0.92	M = 3.91, SD = 0.94	M = 3.84, SD = 0.97	.228	.796	.00
Prosocial Behavior	M = 3.24, SD = 0.58	M = 3.24, SD = 0.60	M = 3.21, SD = 0.59	.135	.874	.00
Mind Perception	M = 2.76, SD = 0.61	M = 2.71, SD = 0.75	M = 2.67, SD = 0.66	.876	.417	.00
Life Satisfaction	M = 4.65, SD = 1.53	M = 4.42, SD = 1.61	M = 4.35, SD = 1.73	2.102	.123	.01

*p < .05. **p < .01.

### Study 1 Discussion

This online study randomized participants (*N =* 625) into one of three emotion conditions––awe (*n* = 220), amusement *(n* = 178), or neutral (*n =* 227)––who then viewed short video clips based on those used by Valdesolo & Graham, (2014) and Piff and colleagues (2015). The awe induction successfully induced awe, as measured by both a single item awe measure (in the PANAS) and a multifactorial measure of awe (the AWE-S), compared to the neutral or amusement conditions. But this did not produce any significant effects in most of the other outcome measures, including supernatural beliefs, intentional pattern perception, uncertainty tolerance, prosocial behavior, and mind perception. Life satisfaction was marginally significantly associated with awe, but only compared to the neutral condition and not the active control.

Under the present conditions, we did not replicate the finding reported in Valdesolo and Graham (2014), Piff and colleagues (2015); and Rudd and colleagues (2012), though it should be noted that they used a few different forms of emotion inductions, especially video but also writing exercises. With Study 1 using video, Study 2 would re-assess using writing exercises.

### Study 2: Writing Manipulations

Study 2 used writing prompts to elicit awe (experimental), pride (control), or neutral (control). The same battery of questionnaires was then administered. According to past research, we hypothesized that the awe manipulation should result in changes to supernatural beliefs, agency detection, prosocial beliefs, and well-being.

## Method

### Participants

A total of 744 participants were recruited using the online survey platform M-Turk. Participants provided written informed consent and agreed that they were over 18 years of age. This study was approved by the first author’s Institutional Review Board.

Of the participants, 174 were removed from the study: 21 participants were removed for failing to respond to the writing task or responding with an irrelevant answer (e.g., writing a random string of letters, copy and pasting text from elsewhere on the survey or internet into the response box); 29 participants were removed for failing the attention check by answering incorrectly when they were asked if they were paying attention to the survey. Lastly, 124 participants were removed for submitting an incomplete survey.

**Participant Characteristics.** In the Awe condition (*n =* 184), the mean age was 35.06 (*SD =* 9.71), 64.1% male, and 82.1% non-Hispanic white, 10.9% Black, and 2.2% Asian or Asian America. In the general positivity condition (*n =* 189), the mean age was 34.38 (*SD =* 10.31), 55.1% male, and 81.0% non-hispanic white, 10.9% black, and 6.3% Asian or Asian American. Finally, in the Neutral condition *(n =* 197), the mean age was 36.08 (*SD =* 11.18), 58.2% male, and 83.2% non-hispanic white, 6.6% black, and 5.6% Asian or Asian American.

## Materials

**Emotion Induction Writing Prompts.** Three emotion induction prompts were used to induce feelings of awe, positivity, or neutral emotion in participants, as adapted from past research (Rudd et al., 2012). Participants were asked to recall circumstances in which they intensely felt the emotion (depending on condition), to try to feel the same feelings while writing about the experience and then responded to items about how they felt during the experience. Prompts were as follows:

*[Awe:] Awe is our emotional response to things perceived to be vast and astonishing that alters the way we understand the world. Please describe a recent experience of awe where nature caused you to feel this way. Try to tell the story exactly as you experienced, from your point of view, in as much detail as possible. Feel free to write as much as you’d like, but please write about 3 sentences.*

*[Positivity:] Please describe a recent experience where you were happy and remember feeling amusement or joy. Try to tell the story exactly as you experienced, from your point of view, in as much detail as possible. Feel free to write as much as you’d like, but please write about 3 sentences.*

*[Neutral:] Please describe your morning routine. Try to tell the story exactly as you experienced, from your point of view, in as much detail as possible. Feel free to write as much as you’d like, but please write about 3 sentences.*

### Measures

The same measures in Study 1 were used. These include a single item awe measure embedded within the PANAS in addition to the AWE-S (which includes vastness, need for accommodation, self-loss, connectedness, time alteration, physiological changes). Additionally, supernatural beliefs, intentional pattern perception, uncertainty intolerance, prosocial behavior, mind perception, and satisfaction with life outcomes were also assessed. Reliabilities for these measures in this sample can be seen in the supplemental materials (S1 Table); however, all the scales and subscales were found to be reliable (α > .70), with the exception of the *Baby* (α = .52) and *Dog* (α = .66) subscales of the Mind Perception Scale.

### Procedure

Participants were randomly assigned to conditions. After the writing prompt, the survey was identical across conditions.

## Results

**Fig 2:**
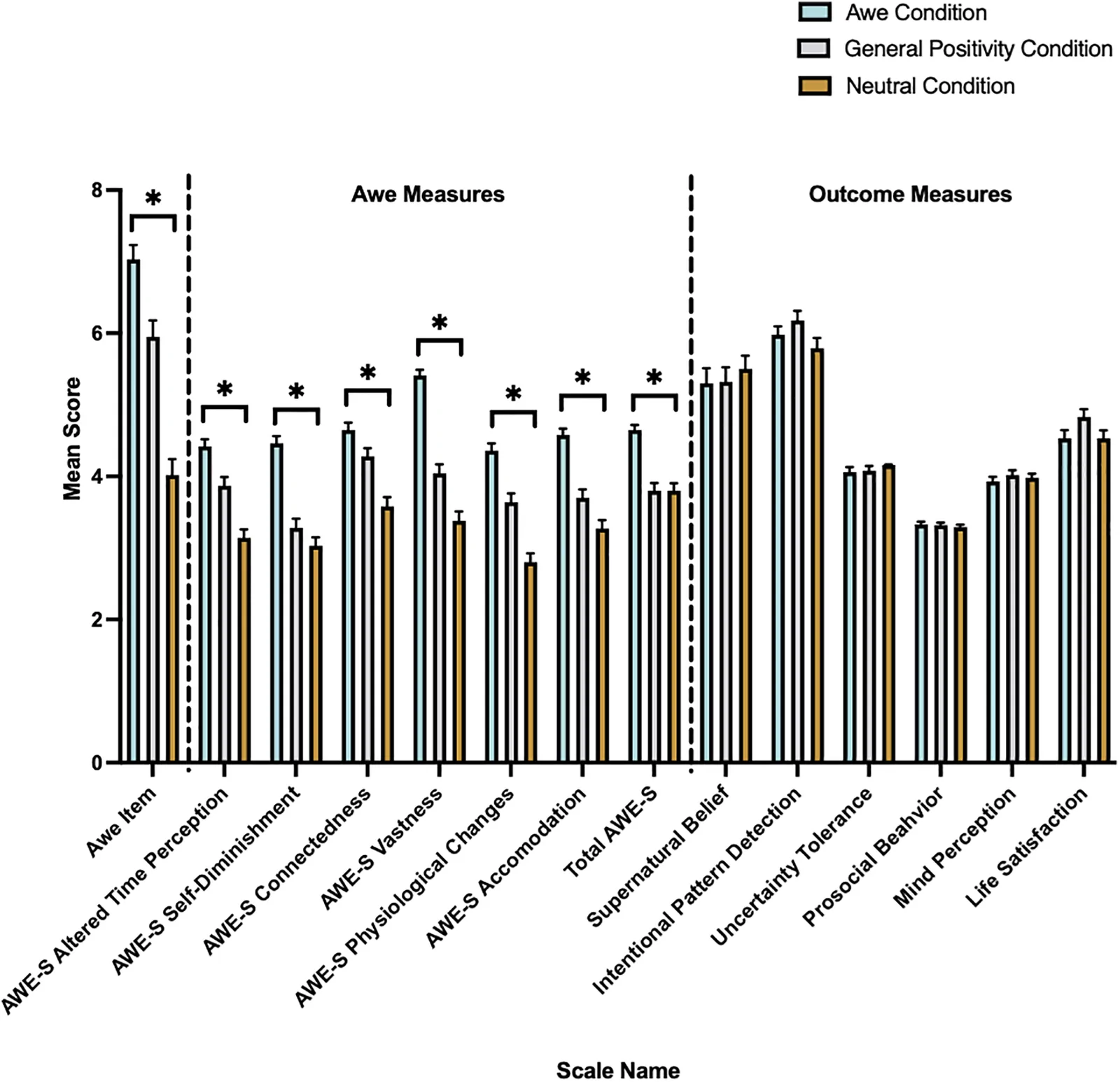
Mean Scores of Awe and Outcome Measures Across Conditions for Study 2. Note: Awe Condition *n =* 184; General Positivity Condition *n =* 189; Neutral condition *n =* 197. The Awe-Item was used as a manipulation check. An asterisk denotes a statistically significant difference at the p < .01 level of omnibus effects between all three conditions. Error bars represents standard error.

**Manipulation Check.** Planned Contrasts revealed that participants reported feeling significantly more awe on the awe item embedded within the PANAS measure in the awe condition (*M* = 7.03, *SD* = 2.74) than the general positivity (*M* = 5.95, *SD* = 3.15) or neutral (*M* = 4.02, *SD* = 3.10) conditions, [*F*(2,569) = 49.454, *p* < .001, *η*_*p*_*²* = .15] (See Fig 2). Scores of positive affect (calculated *without* the embedded awe item) were significantly higher in the general positivity condition (*M* = 7.55, *SD* = 2.28) than the awe condition (*M* = 6.73, *SD* = 2.15) and least in the neutral condition (*M* = 6.17, *SD* = 2.41), [*F*(2, 569) = 18.658, *p* < .001, *η*_*p*_*²* = .03]. There were no significant differences in negative affect between groups.

**Outcome measures.** Planned Contrasts revealed that participants scored significantly higher on awe as measured by the AWE-S total in the awe condition (*M* = 4.65, *SD* = 0.93) than in the neutral (*M* = 3.20, *SD* = 1.58) or general positivity (*M* = 3.80, *SD* = 1.48) conditions, [*F* = [2],569) = 53.763, *p* < .001, *η*_*p*_*²* = 16]. The awe item from the PANAS scale and the total AWE-S score were found to be significantly correlated (*r* = .60, *p < .*001). Furthermore, participants scored significantly higher on each of the dimensions of awe measured by the AWE-S factors (i.e., altered perception of time, connectedness, self-diminishment, vastness, physiological changes, accommodation, and connectedness) in the awe condition than in either the amusement or neutral conditions (these means, and standard deviations are reported in Table 6. There were no significant differences across outcomes (see Table 7; more detail reported in S2 File).

**Table 6:** Group differences in factors of the awe experience scale in study 2.

Dimension of Awe	Awe Condition (N = 184)	General Positivity Condition (N = 189)	Neutral Condition (N = 197)	*F*	*p*	*η*_*p*_ *²*
Altered Perception of Time	M = 4.42, SD = 1.34	M = 3.87, SD = 1.66	M = 3.14, SD = 1.68	31.987	<.001*	.10
Self-Diminishment	M = 4.46, SD = 1.40	M = 3.28, SD = 1.75	M = 3.03, SD = 1.67	41.879	<.001*	.13
Connectedness	M = 4.65, SD = 1.36	M = 4.28, SD = 1.56	M = 3.59 SD = 1.75	22.593	<.001*	.07
Vastness	M = 5.41, SD = 1.08	M = 4.04, SD = 1.75	M = 3.38, SD = 1.83	78.869	<.001*	.22
Physiological Changes	M = 4.36, SD = 1.36	M = 3.65, SD = 1.67	M = 2.80, SD = 1.75	45.429	<.001*	.14
Accommodation	M = 4.58, SD = 1.19	M = 3.70, SD = 1.65	M = 3.27, SD = 1.68	35.821	<.001*	.11
Total AWE-S	M = 4.65 SD = 0.93	M = 3.20 SD = 1.58	M = 3.80 SD = 1.47	53.763	<.001*	.16

*p < .01.

**Table 7:** Group differences across outcomes in study 2.

Outcome	Awe Condition (N = 184)	Amusement Condition (N = 189)	Neutral Condition (N = 197)	*F*	*p*	*η*_*p*_ *²*
Supernatural Belief	M = 5.30, SD = 2.85	M = 5.32, SD = 2.78	M = 5.5 SD = 2.62	.510	.601	.00
Intentional Pattern Perception	M = 5.98, SD = 1.57	M = 6.18, SD = 1.82	M = 5.79, SD = 2.03	2.197	.112	.01
Uncertainty Tolerance	M = 4.06, SD = .94	M = 4.08, SD = .90	M = 4.16, SD = 0.06	.696	.499	.00.
Prosocial Behavior	M = 3.33, SD = .49	M = 3.32, SD = .46	M = 3.29, SD = 0.49	.312	.732	.00
Mind Perception	M = 3.93, SD = 0.86	M = 4.02, SD = 0.89	M = 3.98, SD = 0.83	.491	.612	.00
Life Satisfaction	M = 4.53, SD = 1.57	M = 4.83, SD = 1.47	M = 4.53, SD = 1.58	1.753	.174	.01

*p < .05. **p < .01.

### Study 2 discussion

This study (N = 570) randomly assigned participants into one of three emotion induction conditions––awe (*n =* 184), general positivity (*n =* 189), or neutral *(n* = 197) ––and then asked them to complete a recall writing task. This writing task successfully induced awe, as measured by a single awe item on the PANAS scale and the AWE-S, as compared to the general positivity and neutral conditions. But, contrary to expectations and recent research (Piff et al., 2015; Rudd et al., 2012; Valdesolo & Graham, 2014), no significant effects were found on any of the outcome measures, including supernatural beliefs, intentional pattern perception, uncertainty tolerance, prosocial behavior, mind perception, and life satisfaction. It could be the case that videos and writing may manipulate awe but may do so too subtly to produce other effects. Other, more immersive techniques may provide stronger subjective effects. Furthermore, it may be that in-person, laboratory studies could amplify the effects of these manipulations. These issues are addressed in Study 3.

### Study 3: Virtual reality manipulation

This next study attempted to use virtual reality to elicit awe in a laboratory setting, with a virtual reality version of Google Earth. In the experimental condition, participants were floating above the building in which the study was taking place and slowly floated upwards so that they could see the city, the state, the country, and, eventually, the entire world. Unlike the past two manipulations, this study tried to create a sense of vastness perceptually and cognitively using a virtual reality program. A battery of questionnaires was then administered, with some additions, as we were hoping to test moderators related to beliefs about the world (measured by a “primal world belief” scale) and feelings of connectedness with other people (using the inclusion of others in the self measure). We expected the awe condition (experimental), as compared to an active control and a neutral control, would result in changes to supernatural beliefs, agency detection, prosocial beliefs, and well-being.

## Method

### Participants

A total of 100 participants were recruited at a top American university from the North East using the SONA system to give undergraduate students course credit for participating in studies. Participants provided written informed consent and agreed that they were over 18 years of age. This study was approved by the Institutional Review Board at the University of Pennsylvania.

Of the participants, 9 were removed from the study for incomplete data.

**Participant Characteristics.** In the Awe condition (*n =* 34), the mean age was 19.91 (*SD =* 1.11), 29.4% male, and 44.1% non-Hispanic white, 8.8% Black, and 38.2% Asian or Asian America. In the active control condition (*n =* 30), the mean age was 19.80 (*SD =* 1.32), 36.7% male, and 44.7% non-Hispanic white, 16.7% black, and 23.3% Asian or Asian American. Finally, in the Neutral condition *(n =* 27), the mean age was 36.08 (*SD =* 11.18), 58.2% male, and 48.1% non-Hispanic white, 3.7% black, and 40.7% Asian or Asian American.

## Materials

**Virtual Reality Programs.** Three virtual reality experiences were created for this study to manipulate awe. In each condition, participants put on a virtual reality headset and were told that they are free to look around their environment in every direction. In the experimental (zoom-out) condition, participants were floating in virtual space over a photorealistic representation of the building in which the session was taking place. They then slowly zoomed out to see the city, state, country, and world. This was meant to mimic the overview effect, which has been found to be associated with awe experience [45].

In the active control (zoom-in) condition the process occurred in reverse–participants started floating in space and viewing the world, and then slowly zoomed in to the building in which the study session was occurring. In the neutral control condition, participants were in a virtual living room with all white walls and furniture, designed to be uninteresting.

### Measures

Reliability of measures used in this study are available in the supplement (S2 Table). All scales and subscales used in this study were reliable (α > .70), except for the *Enticing* world belief subscale (*α* = .69) of the primal world belief measure.

**Mood Direction.** Items from the Positive and Negative Affect Scale developed by Watson, Clark, and Tellegen (1988) measured both positive and negative emotion. For Negative Affect, participants were presented with statements such as “In this moment I feel anxious,” and then asked to evaluate the degree to which they felt the given emotion. As in the previous studies, a single-item measure of awe was embedded within the PANAS.

**Awe.** The Awe Experience Scale (AWE-S; Yaden et al., 2019) was administered. This scale measures various qualities associated with awe experience and contains six subscales: vastness, accommodation, self-diminishment, connectedness, time alteration, and physiological reactions.

**Presence**. The sense of presence, or of feeling ‘present’ in the virtual environment, were measured by the ITC-Sense of Presence Inventory [46]. Sample item “I had a sense of “being there” in the virtual space.”

**Inclusion of Other in Self.** The Inclusion of Other in Self (IOS; Aron et al., 1992) [47] item was administered to measure the degree to which participants felt connected to other people. The item features two circles, one marked ‘self’ and one marked ‘other’ that are more or less overlapping. Participants indicate how connected to others they feel by indicating less or more overlapping circles.

**Intentional Pattern Perception.** Intentional Pattern Perception was measured using the same numerical judgment task from Study 1 adopted from past research by Valdesolo and Graham (2014).

**Primal World Beliefs**. The Primals Inventory [48] is a measure of beliefs about the world as a whole. In this case, four sub-scales were used: Interconnectedness (e.g., “The world is a place where everything is completely interconnected”), Safe (e.g., “I tend to see the world as pretty safe”), Enticing (“No matter where we are, incredible beauty is always around us”), and Alive (“What happens in the world in meant to happen”).

**Supernatural Belief.** Supernatural Belief was measured using questions adopted from past research by Valdesolo and Graham (2014). Given that supernatural control (i.e., the idea that the universe is controlled by God or a supernatural force) was the measure of interest in past research by Valdesolo and Graham (2014), the three-item index of belief in supernatural control [38] was also scored separately.

**Prosocial Behavior.** We used Piff and colleagues (2015) task measure of prosocial behavior in which a research assistant pretends to drop several pens on the floor and then counts how many pens the participant helps pick up.

**Life Satisfaction.** Given that Rudd, Vohs, and Aker (2012) used The Satisfaction with Life Scale (SWLS) developed by Diener et al. (1985), we used SWLS to measure life satisfaction.

### Procedure

After receiving the experimental manipulation, participants filled out the same questionnaire.

## Results

**Fig 3:**
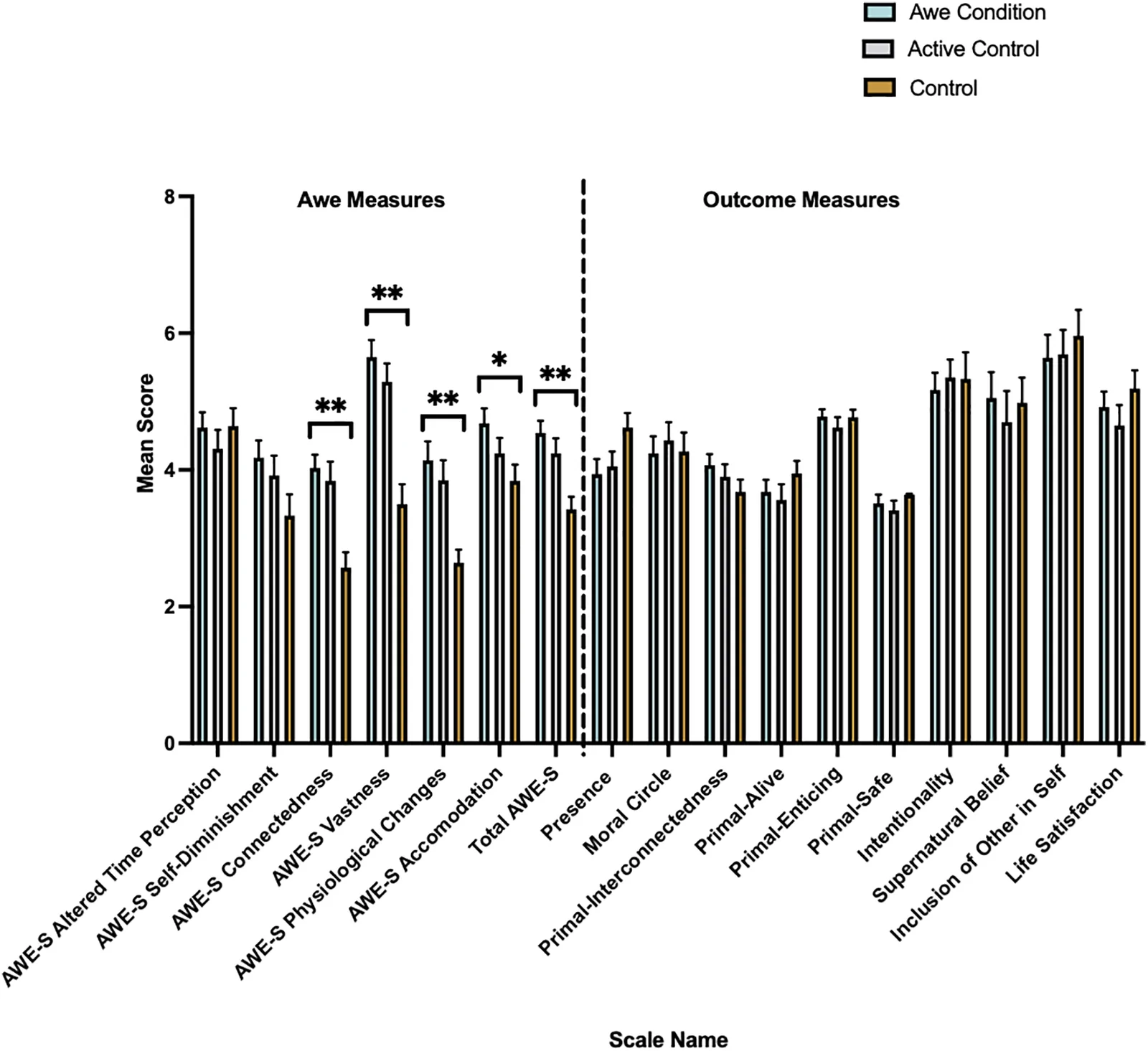
Mean Scores of Awe and Outcome Measures Across Conditions for Study 3. Note: The Moral Circle mean scores were divided by ten to visually represent them alongside the other measures. An asterisk denotes a statistically significant difference at the p < .05 level and a double asterisk denotes a statistically significant difference at the p < .01 level.

**Manipulation Check.** Planned contrasts revealed that participants reported feeling significantly more awe on the awe items embedded within the PANAS measure in the experimental condition (M = 7.512, SD = 2.63) and the active control (M = 7.21, SD = 2.22) than the neutral control (M = 5.52, SD = 2.50) conditions [*F*(2, 86) = 4.183, *p* < .018, *η*_*p*_*²* = .09] (see Fig 3). Tukey’s post-hoc tests showed that the experimental condition and active control were not different from one another, but both were significantly higher than the neutral control.

**Outcome measures.** Though awe increased in the experimental condition, there were no significant differences in outcomes. Planned contrasts revealed that participants scored higher on awe as measured by the AWE-S total in the experimental condition (*M* = 4.54, *SD* = 1.05) and the active control (*M* = 4.24, *SD* = 1.22) than the neutral control (*M* = 3.42, *SD* = .98) [*F*(2, 88) = 8.166, *p* < .001, *η*_*p*_*² = .16*]. Tukey’s post-hoc tests showed that the experimental condition and active control were not different from one another, but both were significantly higher than the neutral control. Additional differences across the sub-scales of the AWE-S are reported in Table 8. There were no significant differences across outcomes (see Table 9; more detail reported in S3 File).

**Table 8:** Group differences in factors of the awe experience scale in study 3.

Dimension of Awe	Zoom Out (N = 34)	Zoom In (N = 30)	Control (N = 27)	*F*	*p*	*η*_*p*_ *²*
Altered Perception of Time	*M* = 4.62, *SD* = 1.29	*M* = 4.31, *SD* = 1.51	*M* = 4.64, *SD* = 1.36	.524	.594	.01
Self-Diminishment	*M* = 4.18, *SD* = 1.46	*M* = 3.92, *SD* = 1.58	*M* = 3.33, *SD* = 1.63	2.289	.107	.05
Connectedness/Unity	*M* = 4.03, *SD* = 1.11	*M* = 3.84, *SD* = 1.54	*M* = 2.57, *SD* = 1.16	10.850	<.001*	.20
Vastness	*M* = 5.65, *SD* = 1.45	*M* = 5.29, *SD* = 1.46	*M* = 3.50, *SD* = 1.52	16.789	<.001*	.28
Physiological Changes	*M* = 4.14, *SD* = 1.62	*M* = 3.85, *SD* = 1.60	*M* = 2.64, *SD* = 1.00	8.618	<.001*	.16
Accommodation	*M* = 4.68, *SD* = 1.28	*M* = 4.24, *SD* = 1.26	*M* = 3.84, *SD* = 1.23	3.314	.041*	.07
AWE-S Total Scale	*M* = 4.54, *SD* = 1.05	*M* = 4.24, *SD* = 1.22	*M* = 3.42, *SD* = 0.98	8.166	.001*	.16

*p < .05. **p < .01.

**Table 9:** Group differences in outcomes across conditions in study 3.

Dimension of Awe	Zoom Out (N = 34)	Zoom In (N = 30)	Control (N = 27)	*F*	*p*	*η*_*p*_ *²*
Presence	M = 3.94, SD = 1.28	M = 4.05, SD = 1.20	M = 4.62 SD = 1.10	2.687	.074	.06
Moral Circle	M = 42.38, SD = 14.66	M = 44.30, SD = 14.55	M = 42.67, SD = 14.41	.156	.856	.00
Primal-Interconnectedness	M = 4.07, SD = .94	M = 3.90, SD = 1.01	M = 3.68, SD = .93	1.281	.283	.02
Primal-Alive	M = 3.68, SD = 1.03	M = 3.56, SD = 1.26	M = 3.95, SD = 0.94	.938	.395	.02
Primal-Enticing	M = 4.78, SD = .63	M = 4.62, SD = .83	M = 4.77, SD = .58	.485	.618	.02
Primal-Safe	M = 3.51, SD = .75	M = 3.41, SD = .76	M = 3.64, SD = .070	.659	.520	.02
Intentionality	M = 5.17, SD = 1.46	M = 5.35, SD = 1.44	M = 5.33, SD = 2.03	.111	.895	.00
Supernatural Belief	M = 5.05 SD = 2.21	M = 4.70, SD = 2.49	M = 4.98, SD = 1.92	.216	.806	.00
Inclusion of Other in Self	M = 5.64 SD = 1.97	M = 5.69 SD = 1.95	M = 5.96 SD = 1.97	.207	.813	.00
Life Satisfaction	M = 4.92, SD = 1.31	M = 4.65, SD = 1.63	M = 5.19, SD = 1.38	.966	.385	.02

*p < .05. **p < .01.

### Study 3 Discussion

This study randomly assigned participants into one of three emotion induction conditions and assessed feelings of awe as well as other outcomes. The virtual reality induction successfully induced awe in the experimental and active control conditions, as measured by a single awe item on the PANAS scale and most sub-scales of the AWE-S, as compared to the neutral control condition. No significant effects were found on any of the outcome measures, including supernatural beliefs, intentional pattern perception, primal world beliefs, prosocial behavior, moral circle, inclusion of other in the self, and life satisfaction. This was true across all three groups as well as between only the experimental and neutral conditions. This study used a slightly more immersive induction technique (virtual reality as opposed to writing or a video) and was conducted in-person in a laboratory setting. It may be the case that these studies were still too subtle manipulations of awe to impact outcome measures. It could also be the case that previous studies on awe found significant effects through random variation in smaller sample sizes.

### Direct replications

The conceptual replications had several notable limitations. First, the battery of outcome measures was delivered in the same order each time to all participants across all three conceptual replication studies. Therefore, order effects may explain null results especially for outcome measures which appeared toward the end of the battery. Second, the potentially fleeting nature of awe may have made it impossible to capture an outcome effect in so large of a battery of outcomes measures in general. Third, some of our manipulations and outcome measures were inspired by, but not exactly the same as, the three studies we based our replications on [3–5].

To address these limitations, we next conducted a series of direct replications of the studies mentioned in Table 1. We chose studies from past research that could be replicated using online methods in order to collect larger sample sizes. Rather than presenting multiple outcome measures following awe induction, we directly replicated the three studies using three distinct samples that contained the same exact materials and presented in the same exact way as the original studies.

For these direct replication studies, the target sample was increased to 2.5 times the original [36]. Finally, though part of the analyses of the original papers, mediation analyses could not be conducted in any of the direct replications in our studies as we found no difference in outcomes between conditions across replications. This set of studies was approved by Johns Hopkin’s University’s Institutional Review Board. These studies are presented below. Preregistration for the series of direct replications is available at: https://doi.org/10.17605/OSF.IO/S9MCN and all data associated with this study have been made available at: https://osf.io/458qc/.

### Study 4: Direct replication of video manipulation

Study 4 in our study was a designed as a direct replication of Valdesolo and Graham’s (2014; Study 1) video manipulation to elicit awe, amusement, or no emotion (neutral). Several authors (M.G., D.B.Y., along an original author, Valdesolo) have previously conducted a pre-registered direct replication using an in-person college campus sample as the authors did in the original study, similarly finding that the awe manipulation was successful, but outcomes from the original study did not replicate (Yaden et al., in press). To increase statistical power and sample size, an online Prolific sample was chosen rather than recruiting participants from a college campus. We acknowledge that this represents a methodological deviation from the original study. However, the participants in our study viewed the exact same video stimuli that the original study participants viewed on iPads, and the measures, procedures, and analytic approach were otherwise matched to those used by the original authors. Consistent with our definition of direct replication (p.7), we classify this study as a direct replication while recognizing that the change may have influenced the findings.

### Participants

A total of 312 participants were recruited using the online survey platform Prolific. Participants provided written informed consent and agreed that they were over 18 years of age.

Of the initial 312 participants, 12 were removed from the study: 3 for failing 1 of the 2 attention checks built into the survey. Lastly, 9 participants were removed for submitting an incomplete survey. A total of 300 participants remained. This is 2.5 times the 120 participants recruited in the original study.

**Participant Characteristics.** In the Awe condition (*N =* 108), the mean age was 42.83 (*SD =* 13.99), 58% female, and 82% non-Hispanic white, 7% Black, and 6% Asian or Asian American. In the amusement condition (*N =* 101), the mean age was 39.55 (*SD =* 13.24), 64% female, and 83% non-Hispanic white, 11% black, and 3% Asian or Asian American. Finally, in the Neutral condition (*N =* 91), the mean age was 40.46 (*SD =* 14.30), 62% female, and 79% non-Hispanic white, 8% black, and 10% Asian or Asian American.

## Materials

**Emotion Induction Videos.** The videos were the same ones that were used in the original study: the awe clip consisted of sweeping views of natural scenery (from the BBC’s *Planet Earth*), the amusement clip was comedic footage of animals (from the BBC’s *Walk on the Wild Side*), and the neutral clip was an interview (a 1959 news interview conducted by Mike Wallace). Participants then answered the same questions that were administered and analyzed in Study 1 of Valdesolo & Graham (2014).

### Measures

**Supernatural control** [38]. This 3-item measure asks participants to rate the extent to which they believe that various supernatural forces exert control over events.

**Belief in God** [49]. This single item is a general measure of belief in God.

**Other supernatural beliefs** [50]. These are four items related to other supernatural beliefs (curses, ghosts, miracles, and angels). These items were included in the original study, but their analysis was not reported. We include them here in order to exactly replicate the original methods but make no predictions about the effect of condition on these items.

**Emotional states.** An 8-item measure of self-reported emotional states, including a manipulation check that asked participants to indicate how much awe they felt during the video. This was an ad-hoc scale created by the authors of the original study.

### Procedure

The survey was created using Qualtrics survey software and distributed on *Prolific*, an online survey platform. Once participants had given their consent to participate, they were randomly assigned to one of the three video conditions: the awe video (experimental), the amusement video (control), or the neutral affect video (control). After watching the video, the rest of the survey was the same for all participants.

## Results

**Manipulation Check.** A one-way ANOVA revealed that there were significant differences in self-reported awe between the Awe condition (*M* = 5.56, *SD =* 1.61), Amusement (*M* = 3.00, *SD =* 1.70) and Neutral conditions (*M* = 4.27, *SD =* 1.63), *F*(2,297)=63.390, *p* < .001, *η*^2^ = .30. Planned contrasts demonstrated that awe was reported to be significantly higher in the Awe condition when compared to the Neutral condition alone (*t*(297)=5.508, *p* < .001), as well as when compared with the Amusement condition alone (*t*(297)=11.257, *p* < .001).

**Fig 4:**
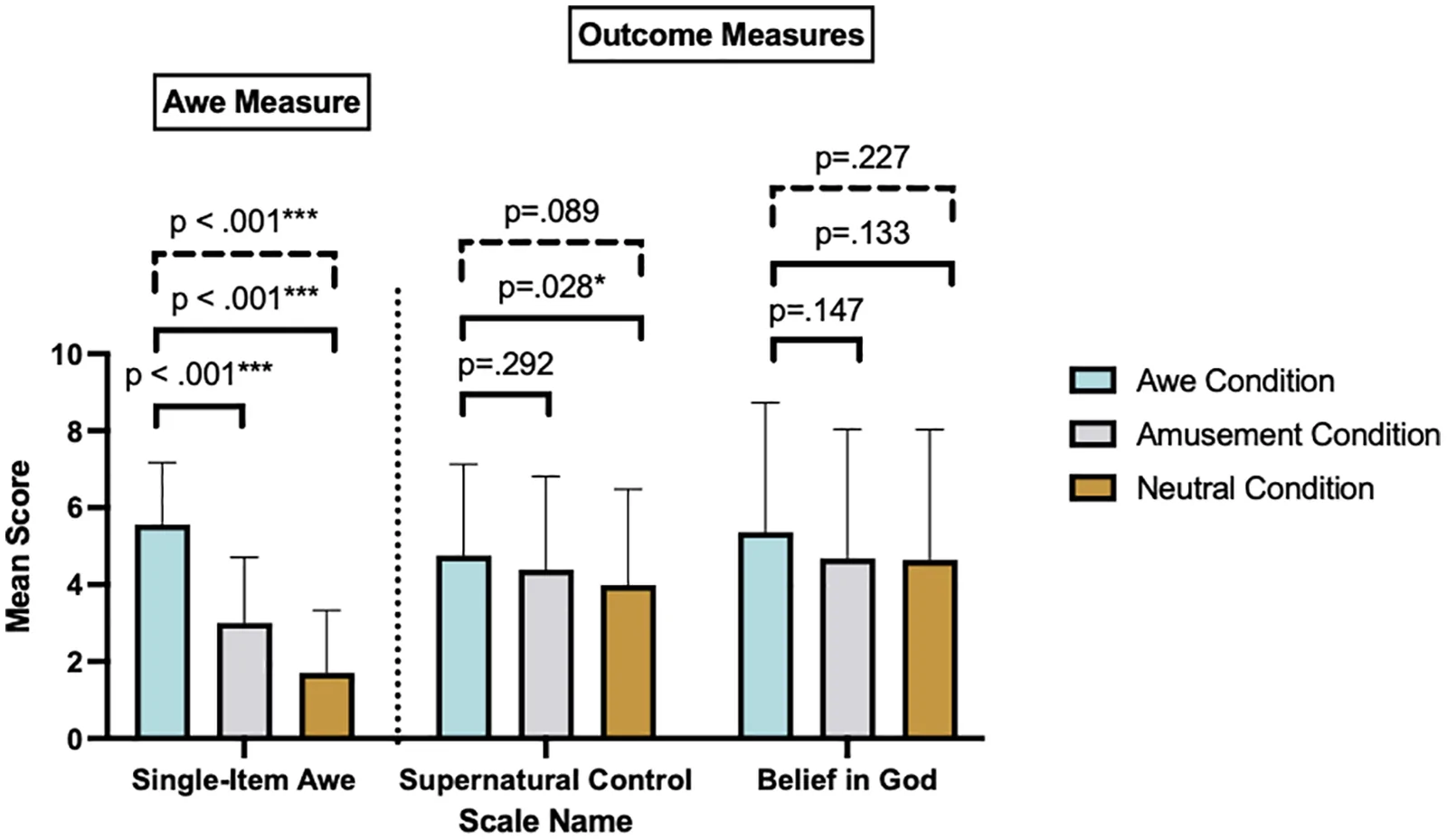
Mean Scores of Awe and Outcome Measures Across Conditions. Note: Awe Condition *n =* 108; Amusement Condition *n =* 101; Neutral Condition *n =* 91. The Awe-Item was used as a manipulation check. A dotted line represents the results of a one-way ANOVA and a solid line represents the results of planned contrasts. Error bars represent standard error.

**Outcome measures.** There was no difference on either outcome measure––supernatural control or belief in God––between the experimental Awe and the control conditions of Amusement and Neutral (see Fig 4).

Ratings of supernatural control did not differ between the Awe (*M* = 4.75, *SD =* 2.38) Amusement (*M* = 4.39, *SD =* 2.42) or Neutral conditions (*M* = 3.99, *SD =* 2.49, *F*(2,297)=2.436, *p* = .089, *η*_*p*_*²* = .02. Planned contrasts demonstrated no difference in supernatural control when the Awe condition was compared to the Amusement condition alone (*t*(297)=1.055, *p* = .292), however, did show statistically significant differences when the Awe condition was compared to the Neutral condition alone (*t*(297)=2.207, *p* = .028).

Ratings of belief in God did not differ between the Awe (*M* = 5.36, *SD =* 3.37) Amusement (*M* = 4.68, *SD =* 3.36) or Neutral conditions (*M* = 4.64, *SD =* 3.39), *F*(2,297)=1.492, *p* = .227, *η*_*p*_*²* = .01.

Other single-item supernatural beliefs (curses, ghosts, angels, and miracles) did not show any statistically significant differences between conditions in planned comparisons. The exploratory ANOVAs conducted on single-item ratings of other supernatural beliefs are available in the supplement (S3 File).

**Mediation Analyses.** The original authors reported that awe mediated the relationship between condition and the supernatural control outcome. In our study, because we did not observe significant effects of condition on the outcome variable, we did not attempt to replicate the mediation analyses reported by the original authors.

### Study 4 Discussion

This online study randomized participants (*N =* 300) into one of three emotion conditions––awe (*n* = 108), amusement *(n* = 101), or neutral (*n =* 91)––who then viewed short video clips used by Valdesolo & Graham, (2014). The awe induction successfully induced awe, as measured by a single item awe measure but this did not produce any significant effects in most of the other outcome measures, including supernatural control or belief in God. When the Awe condition was compared to the neutral condition alone there was a significant difference in supernatural control between conditions, though this difference did not hold when compared to the active control condition. This pattern of findings is virtually identical to those found by Yaden et. al. (in press) which conducted a preregistered direct replication (PDR) of this same exact study – using an in-person (*N =* 299) sample.

### Study 5: Direct Replication of Writing Manipulation

Our Study 5 was a direct replication of Piff et al.‘s (2015) Study 2 which used writing and recall prompts to elicit awe (experimental), pride (control), or neutral (control). Like the original study we expected that participants would report a significantly increased small sense of self in the awe condition and that this small self would mediate changes in ethical decision making.

## Method

### Participants

A total of 188 participants were recruited using the online survey platform Prolific. Participants provided written informed consent and agreed that they were over 18 years of age.

Of the 212 participants recruited, 24 were removed from the study: 1 participant was removed for failing to provide consent; 2 participants were removed for failing one of the two attention checks. Lastly, 21 participants were removed for submitting an incomplete survey. A total of 188 participants remained, which is 2.5 times the 75 participants recruited in the original study.

**Participant Characteristics.** In the Awe condition (*n =* 55), the mean age was 39.96 (*SD =* 13.43), 49.1% male, and 42% non-Hispanic white, 10.9% Black, 7.3% Asian or Asian America, and 1.8% Multiracial. In the Pride condition (*n =* 58), the mean age was 40.31 (*SD =* 14.29), 43.1% male, and 45% non-Hispanic white, 10.3% black, and 6.9% Asian or Asian American, and 5.1% Multiracial. Finally, in the Neutral condition *(n =* 75), the mean age was 39.31 (*SD =* 11.99), 22.7% male, and 57% non-Hispanic white, 4% black, 8.0% Asian or Asian American, and 5.3% Multiracial.

## Materials

**Emotion Induction Writing Prompts.** Three emotion induction prompts were used to induce feelings of awe, pride, or neutral emotion in participants, as used in past research (Piff et al., 2015). Participants were asked to recall circumstances in which they intensely felt the emotion (depending on condition). Prompts were as follows:

*[Awe:] Please take a few minutes to think about a particular time, fairly recently, when you encountered a natural scene that caused you to feel awe. This might have been a sunset, a view from a high place, or any other time you were in a natural setting that you felt was beautiful.*

*[Pride:] Please take a few minutes to think about a particular time, fairly recently, when you felt pride. This might have been being accepted to a university, winning an event or competition, or any other time that you achieved a personal accomplishment.*

*[Neutral:] Please take a few minutes to think about something you did fairly recently. This might have been riding a bike, studying for a test, or any other thing that happened during your day.*

### Measures

**Emotion.** Participants were asked to evaluate the degree to which they felt the following emotions on a 7-point Likert-type scale (1 = not at all, 7 = extremely): Anger, Awe, Disgust, Fear, Pride, Sadness, and Happiness.

**Small Self (Shiota et al., 2007).** To measure the small self, participants were asked to evaluate their endorsement of the following prompt on a 7-point Likert-type scale (1 = not at all true, 7 = very true): “I felt the presence of something greater than myself”.

**Ethical Decision Making Task (Detert et al., 2008).** Participants were presented with eight hypothetical scenarios describing self-interested acts that violated social norms and standards of behavior such as:

*“You’ve waited in line for 10 min to buy a coffee and muffin at Starbucks. When you are a couple of blocks away, you realize that the clerk gave you change for $20 rather than for the $10 you gave him. You savor your coffee, muffin, and free $10”.*

For each of the scenarios participants were asked to evaluate how likely they would be to engage in the behavior described on a 7-point Likert-type scale (1 = not at all likely, 7 = highly likely).

### Procedure

Participants were randomly assigned to the awe, pride, or neutral writing task. After the writing prompt participants completed measures of emotion states, followed by the small self item. Participants were then informed that they would complete an unrelated study of filler items which asked about their beliefs about the world. Finally, the ethical decision-making task was administered.

## Results

**Manipulation Check.** A one-way ANOVA revealed that there were significant differences in self-reported awe between the Awe condition (*M* = 5.82, *SD =* 1.26), Pride (*M* = 4.07, *SD =* 2.06) and Neutral conditions (*M* = 2.84, *SD =* 2.00), *F*(2,185)=41.329, *p* < .001, *η*_*p*_*²* = .31. Planned contrasts demonstrated that awe was reported to be significantly higher in the Awe condition when compared to the Neutral condition alone (*t*(185)=5.506, *p* < .001), as well as when compared with the Amusement condition alone (*t*(185)=9.091, *p* < .001).

**Fig 5:**
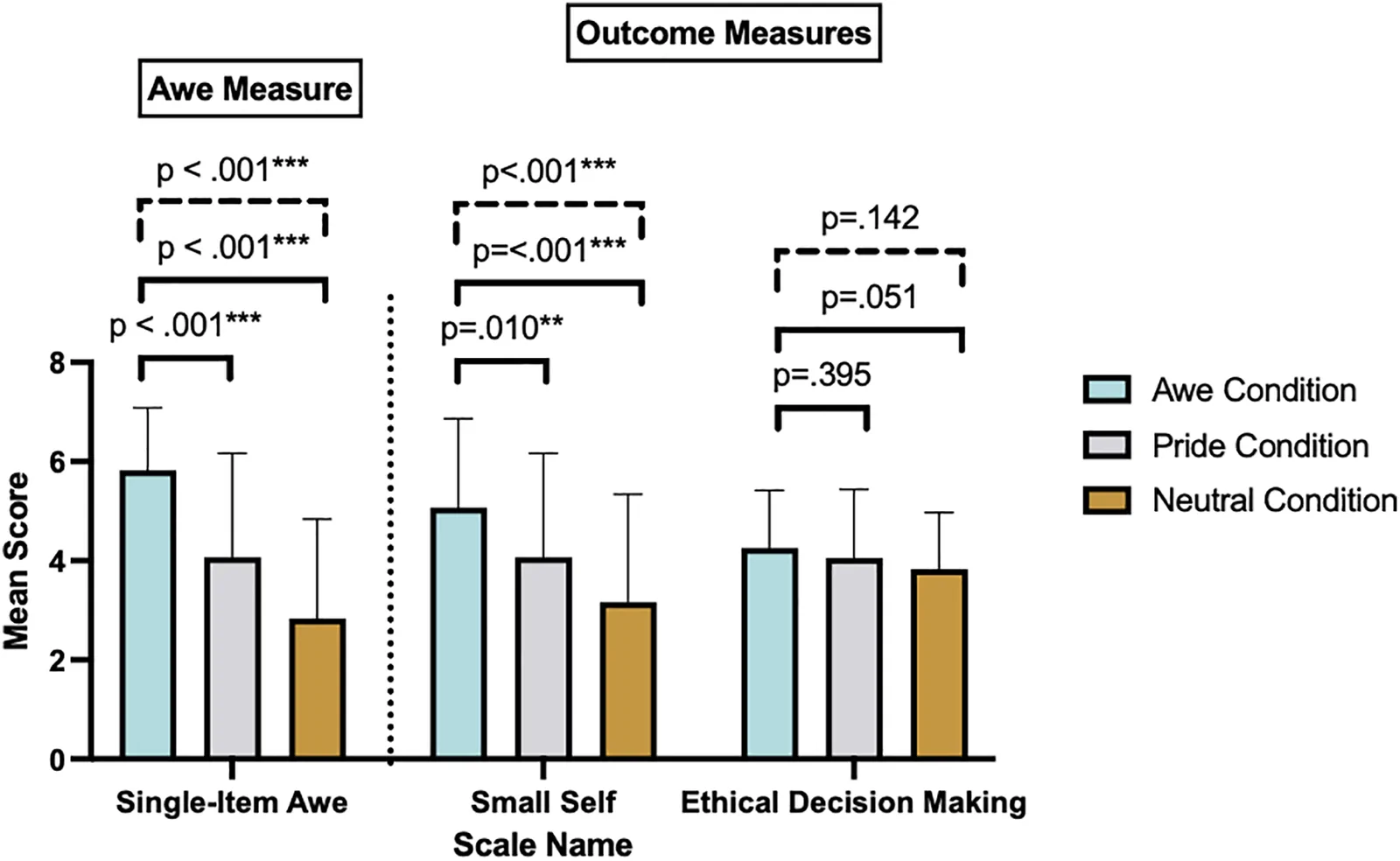
Mean Scores of Awe and Outcome Measures Across Conditions. Note: Awe Condition *n =* 55; Pride Condition *n =* 58; Neutral Condition *n =* 75. The Awe-Item was used as a manipulation check. A dotted line represents the results of a one-way ANOVA and a solid line represents the results of planned contrasts. Error bars represent standard error.

**Outcome measures.** There was a statistically significant difference in Small Self ratings between the Awe, Pride, and Neutral conditions, however, no significant difference between ethical decision making between conditions (see Fig 5).

Ratings on a single small self item did significantly differ between the Awe (*M* = 5.07, *SD =* 1.79) Pride (*M* = 3.16, *SD =* 2.09) and Amusement conditions (*M* = 3.99, *SD =* 2.18), *F*(2,185)=13.930, *p*=<.001, *η*_*p*_*²* = .13. Planned contrasts demonstrated significant differences in small self when the Awe condition was compared to the Pride condition alone (*t(*185)=2.608, *p* = .010) and when the Awe condition was compared to the Neutral condition alone (*t*(185)=5.269, *p*=<.001).

Ratings of ethical decision making did not differ between the Awe (*M* = 4.26, *SD =* 1.16), Pride (*M* = 4.06, *SD =* 1.38) or Neutral conditions (*M* = 3.83, *SD =* 1.14), *F*(2,185)=1.970, *p* = .142, *η*_*p*_*²* = .02.

**Mediation Analyses.** The original authors reported that a small sense of self mediated the relationship between condition and ethical decision-making outcome. In our study, because we did not observe significant effects of condition on the outcome variable, we did not attempt to replicate the mediation analyses reported by the original authors.

### Study 5 Discussion

This study (*N* = 187) randomly assigned participants into one of three emotion induction conditions––awe (*n =* 55), pride (*n =* 58), or neutral *(n* = 75) ––and then asked them to complete a recall writing task. This writing task successfully induced awe, as measured by a single awe item. Like the original study, we also found that ratings on a single-item small self differed between groups, significantly higher in the Awe condition. Finally, unlike the original, we did not find any significant differences between conditions on the ethical decision-making task. Though we can say that we were able to partially replicate Piff et al.‘s (2015) study, it is worth noting that a small sense has been conceptualized as a facet rather than an outcome of awe experience [11,12]. Therefore, it could be argued that the small self-rating in this study serves more as a further manipulation check of a specific facet of awe, rather than as an outcome. On the other hand, the prosocial behavior outcome of interest in this study, ethical decision making, was not significantly different between conditions.

### Study 6: Direct Replication of Story Task

Our study 6 was a direct replication of Rudd et al.‘s (2012; Study 3) study which assigned participants to an awe (experimental) or neutral (control) condition. This manipulation asked participants to imagine themselves as the subjects of a story and then measured the impact of awe on perceived time availability as well as the preference for experiential over material goods. Like the original, we expected the awe condition to induce an expanded feeling of time as well as a preference for experiential (over material) goods.

## Method

### Participants

A total of 268 participants were recruited using the online survey platform Prolific. Participants provided written informed consent and agreed that they were over 18 years of age.

Of the 268 participants recruited, 5 were removed from the study. One participant was removed for failing one of two attention checks and 4 were removed for submitting a blank survey. A total of 263 participants remained, which is 2.5 times the original 105 participant recruited for the original study.

**Participant Characteristics.** In the Awe condition (*n =* 142), the mean age was 38.70 (*SD =* 12.96), 38.7% male, and 83.8% non-Hispanic white, 3.5% Black, 4.2% Asian or Asian American, and 3.5% Multiracial. In the Neutral condition *(n =* 121), the mean age was 38.64 (*SD =* 13.10), 42.1% male, and 83.5% non-Hispanic white, 5.0% black, 6.6% Asian or Asian American, and 0.8% Multiracial.

## Materials

Two emotion induction story prompts were used to induce feelings of awe or neutral emotion in participants, as used in past research [5]. Participants were asked to imagine themselves participating in a story either about climbing the Eiffel tower (Awe condition) or a story about ascending an unnamed tower (Neutral condition). Prompts are in the Supplement (S4 File).

### Measures

**Emotion.** Participants were asked to rate their current feelings of the following emotions on a 7-point Likert type scale (1 = not at all, 7 = very much): calmness, anxiety, relaxation, worry, awe, sadness, boredom, and fear.

**Perceived Time Availability.** Perceived time availability was measured using a 4-item index created for the purposes of the original study which contained items such as “I have lots of time in which I can get things done,” “Time is expanded”, “Time is boundless” and “Time is slipping away” (which was a revere-scored item. Participants were asked to evaluate these prompts on a 7-tiem Likert-type scale.

**Life Satisfaction (Kahneman et. al, 2006).** Participants were asked to fill out a single-item measure of momentary life satisfaction (“All things considered, how satisfied are you with your life as a whole, right now?”) from 1 = not at all satisfied with life to 7 = extremely satisfied with life.

**Experiential vs. Material Goods.** Participants were asked to make hypothetical choices between material goods (such as a watch) and experiential goods (such as Broadway show tickets) said to be equivalent in price.

### Procedure

Participants were told that they would be completing several unrelated surveys. First participants were instructed to read the story task (to which they were randomly assigned an awe story or a neutral story). Next, participants completed filler items followed by a perceived time availability index. The momentary life satisfaction item was administered next, followed by the material vs. experiential goods task. Finally, single-item measures of various emotion states were given.

## Results

**Manipulation Check.** Following the analyses of the original study a one-way ANOVA was conducted to compare the means between the two groups on the single-item measure of awe (and other emotion states). The ANOVA revealed that participants did *not* report feeling significantly more awe on a single-item awe rating in the experimental Awe condition (M = 2.76, SD = 1.39) versus the Neutral control (M = 2.48, SD = 1.34) condition, *F*(1, 261) = 2.774, *p* = .097, *η*_*p*_*² = .01*.

**Fig 6:**
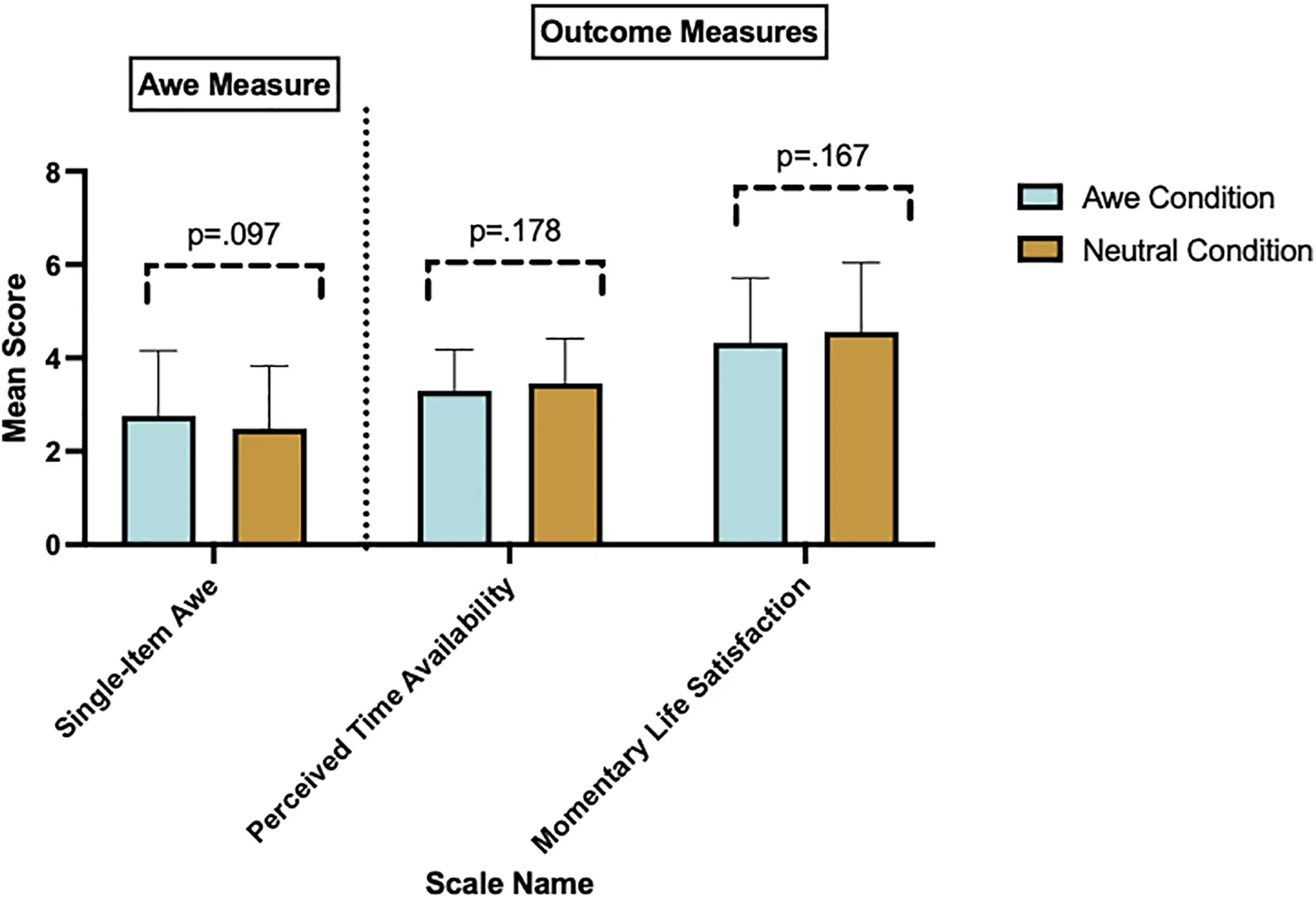
Mean Scores of Awe and Outcome Measures Across Conditions. Note: Awe Condition *n =* 142; Neutral Condition *n =* 121. The Awe-Item was used as a manipulation check. A dotted line represents the results of a one-way ANOVA and a solid line represents the results of planned contrasts. Error bars represent standard error.

**Fig 7:**
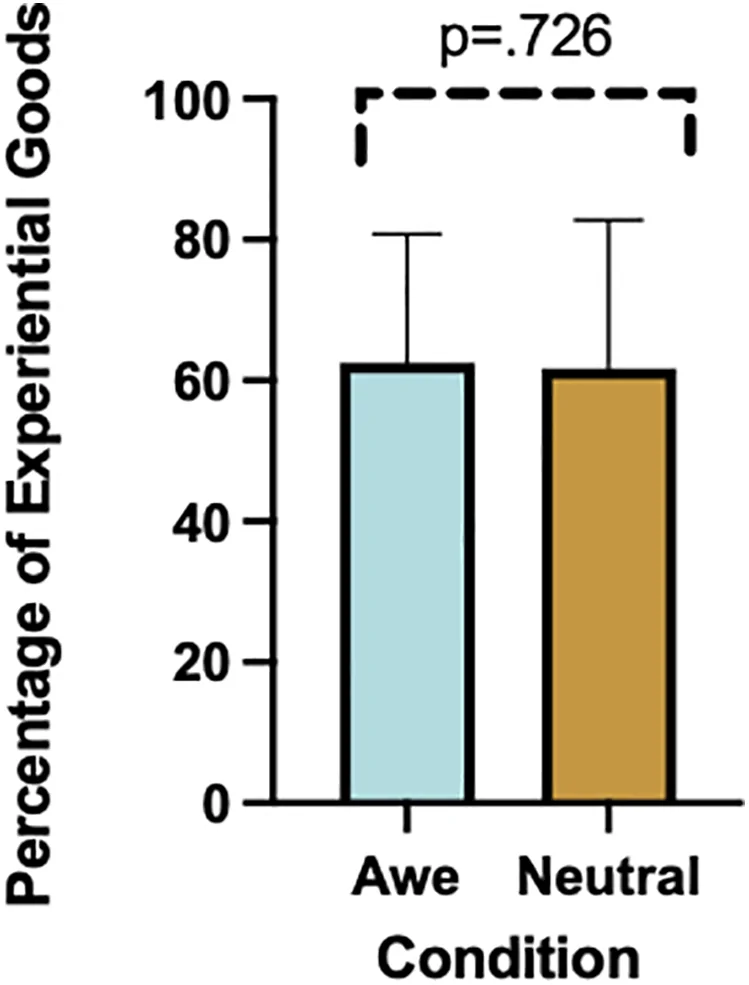
Percentage of Preference for Experiential Goods by Condition. Note: Awe Condition *n =* 142; Neutral Condition *n =* 121. A dotted line represents the results of a one-way ANOVA. Error bars represent standard error.

**Outcome measures.** Following the original analyses, one-way ANOVAs were conducted to compare means between the conditions for each of the outcome measures. There was no difference on either outcome measure––perceived time availability, preference for experiential goods, or momentary life satisfaction––between the experimental Awe and the control condition. These differences in awe, perceived time availability, and momentary life satisfaction are presented in Figs 6 and due to the vastly different scale the mean percentage of preference for experiential vs. material goods between conditions is presented in Fig 7.

Ratings of perceived time availability did not differ between the Awe (*M* = 3.29, *SD =* .89) or Neutral conditions (*M* = 3.45, *SD =* .96), *F*(1,261)=.124, *p* = .726, *η*_*p*_*²* = .01.

The mean percentage of preference for experiential goods did not differ between the Awe (*M* = 62.56, *SD =* 18.21) or Neutral conditions (*M* = 61.71,*SD =* 21.04), *F*(1,261)=1.823, *p* = .187, *η*_*p*_*²* = .00.

Ratings of momentary life satisfaction did not differ between the Awe (*M* = 4.32,*SD =* 1.39) or Neutral condition (*M* = 4.56, *SD =* 1.48), *F*(1,261)=2.774, *p* = .097, *η*_*p*_*²* = .01.

**Mediation Analyses.** The original authors reported that perceived time availability mediated the relationship between condition and the outcomes of momentary life satisfaction and preference for experiential over material goods. In our study, because we did not observe significant effects of condition on either outcome variable, we did not attempt to replicate the mediation analyses reported by the original authors.

### Study 6 Discussion

This online study randomized participants (*N =* 300) into one of two emotion conditions––awe (*n* = 108), amusement *(n* = 101), or neutral (*n =* 91)––who then were asked to read a story and imagine themselves either climbing the Eiffel tower or climbing an unnamed tower [5]. Unlike the other studies in which awe was successfully induced but predicted outcomes did not change, here the awe induction did not successfully induce awe (and outcomes again did not change). Since the awe induction was unsuccessful, it is unclear whether the lack of effects on the outcomes was due to the lack of such an effect or due to the failure of the awe induction.

## General Discussion

Findings in this paper suggest that several published studies on the impact of awe on specific outcomes did not replicate. Across the first five awe induction studies, but not Study 6, awe was successfully induced (in a video task in Study 1 and Study 4; a writing recollection task in Study 2 and study 5; and virtual reality in Study 3; see Fig 1). In all 6 studies, we failed to replicate previously observable changes in outcomes (namely, supernatural control, intentional pattern perception, uncertainty tolerance, perceived time availability, life satisfaction, ethnical decision making and prosocial tendencies). Although experimental manipulations largely failed to produce differences between conditions, exploratory correlations indicated that individuals who reported stronger experiences of awe tended to score higher on several outcomes. Though causal conclusions cannot be drawn, these analyses raise the possibility that the magnitude or intensity of the awe experience may be an important feature to examine in future research.

These findings corroborate emerging research casting doubt on the replicability of awe-induction studies. Elk & Rottevel (2020) found that awe induction did not affect time perception as suggested by past research. In a pre-registered direct replication of Valdesolo and Graham’s Study 1 (Yaden et al., in press), the authors again demonstrated that though awe was successfully induced there were no differences in conditions (awe, amusement, and neutral) for the outcome measures of supernatural control and other supernatural beliefs in an in-person study. In Yaden et al. (in press) Bayesian analyses complemented frequentist analyses to examine the null effects from various angles. Finally, work on awe’s influence on theistic beliefs (Valdesolo et al., in prep) also failed to produce any difference in outcomes following awe induction.

In the present series of studies, most of the findings were null with very few exceptions. Of 6 studies with 33 total outcomes, only a few were significant. In the conceptual (non-direct) replications there was one significant outcome. In the direct replications there were two significant outcomes. These exceptions are described below. In Study 1, the video task was able to produce a significant effect on well-being when the awe condition was compared only to the neutral condition. This partially supported past research conducted by Rudd, Vohs, and Aaker (2012), which found that awe led to feelings of increased life satisfaction. Still, in the direct replication of this association (Study 6), this significant relationship went away. Additionally, in Study 2, the writing task was not able to reproduce this outcome of increased feelings of life satisfaction and awe. Similarly, in Study 4, a video task was able to produce significant differences in supernatural control when the awe condition was compared solely with the neutral condition. In a pre-registered direct replication using an in-person sample (PDR; Yaden et al., in press), which used a similar sized sample (N = 296), neither frequentist analyses nor Bayesian analyses replicated the significant effect of awe on supernatural control when looking only at the awe and neutral conditions. Finally, in Study 5, there was a statistically significant effect of awe small-self ratings, which replicated past research by Piff et al. (2015).

Because many awe studies rely on a single stimulus induction, some sample-specific variability may remain. We attempted to mitigate this concern by employing different sampling methods across studies. The studies in this paper also employed four different emotion induction methods, watching videos (Study 1 and 4), writing about a previous experience (Study 2 and 5), virtual reality (Study 3), and a story task (Study 6). All induction methods except for the story task successfully induced awe, as measured by a single awe item (that uses the term “awe”) in Studies 1–5 and a more robust, multi-dimensional state measure of awe that does not mention the word “awe” in Studies 1–3 [12]. The video, used in previous research by Valdesolo and Graham (2014), involved time-lapse footage of Yosemite State Park, which fit Keltner and Haidt’s criteria for perception of vastness. Participants were later asked to describe something about the video to ensure that they watched the video. The writing task, adapted from previous research by Rudd, Vohs, and Aaker (2012) and Piff and colleagues (2015), asked participants to provide their own experience of awe. The written accounts were checked to ensure that participants were following instructions and wrote something appropriate. Finally, virtual reality was used to simulate participants’ experience of zooming in and out of a photorealistic depiction of the building the study took place in.

The issue with the emotion inductions that we used appears to be less a matter of if they worked––as awe was significantly increased across both awe measures (except for the story Task in Study 6)––but rather to what *magnitude*. That is, how intense was the experience of awe induced by these methods? Taken together, these studies underscore the concern that the intensity of awe induced may be an important factor in eliciting the various sequalae of awe that have been reported in previous studies [20]. Awe that is too subtle may not result in other psychological or physiological changes. It may be the case that more profound and sustained awe experiences (e.g., psychedelic use; [8,16,48,51]) or religious ritual [52,53] have a bigger impact that laboratory manipulations. For instance, in a cross-sectional survey where participants were asked to recall their naturalistic psychedelic experiences, awe (as measured by the short form of the Awe Experience Scale; AWS-SF) correlated with life satisfaction and psychological richness [11]. There may be an interesting parallel between “micro” dosing and “macro” dosing of psychedelics and the intensity of awe in emotion induction contexts (i.e., “micro” and “macro” doses of awe).

The samples used in this study were substantially higher than the sample sizes used in the studies that we failed to replicate both in the conceptual replications and the direct replications. This suggests the possibility that the noisier data in the previous smaller samples may have created Type 1 errors. There were, however, several limitations to our studies. Study 1 and 2 utilized online samples gathered through Amazon’s Mechanical Turk. These participants may have been less motivated than the students and passersby recruited from college campuses in the previous studies. However, the PDR conducted by Yaden and colleagues (in press) also recruited from a college campus and were unable to replicate the association between awe and supernatural control. Furthermore, while manipulation checks were employed in online studies, individuals may have consciously chosen to engage with the study to the bare minimum degree to collect the survey fee. We ran an in-person laboratory-based study to address this issue (Study 3), but we obtained the same results.

## Conclusion

Awe has often been cast as an overwhelming response to vastness and resulting in sometimes lasting psychological changes. These six experimental studies used some of the most common emotion awe-induction methods from across the literature, which successfully increased awe (in all but one study), but we observed no change in outcomes (supernatural control, supernatural beliefs, intentional pattern perception, uncertainty intolerance, perceived time availability, ethical decision making, prosocial tendencies, or mind perception), although in one case we observed an increase in life-satisfaction. Future research might explore whether brief online inductions may not be sufficient to measure the outcomes of awe (as these may be “micro” doses of awe), and more substantive induction methods may be required (i.e., “macro doses”) Additionally, rather than focus solely on statistical significance, future work might also examine the magnitude of these inductions more closely as more intensive manipulations of awe may be necessary to observe effects. Additionally, researchers could look to study awe using elicitors that more reliably induce larger shifts in awe that can be sustained long enough to study its effects as well as the experience of awe itself.

## Supporting information

S1 Table Reliability Analyses for Studies 1 and 2. (DOCX)

S2 Table Reliability Analyses for Study 3. (DOCX)

S3 Table Correlations between Awe Item, Awe-S, Small Self and Primary Outcome Variables. (DOCX)

S1 File Study 1 – Findings Across Outcome Measures. (DOCX)

S2 File Study 2 – Findings Across Outcome Measures. (DOCX)

S3 File Study 3 – Findings Across Outcome Measures. (DOCX)

S4 File Study 6 – Emotion Induction Prompts. (DOCX)
