# Supplementary material for: A series of replication attempts of experimental awe-induction effects
Source: PLoS One. 2026 Sep 2;21(9):e0356728. doi: 10.1371/journal.pone.0356728 (PMC13537573; doi:10.1371/journal.pone.0356728)
Supplement: Supplementary file 1 [file pone.0356728.s001.docx]

**Supplemental Materials**

A Series of Replication Attempts of Experimental Awe-Induction Effects

**S1 Table**

*Reliability Analyses for Studies 1 and 2*

| Scale | Subscale | No. of Items | Range | Reliability  Study 1 | Reliability  Study 2 |
| --- | --- | --- | --- | --- | --- |
| PANAS | Negative Affect | 6 | 1-11 | α = .88 | α = .93 |
| PANAS | Positive Affect | 4 | 1-11 | α = .85 | α = .80 |
| AWE-S | Total Scale | 30 | 1-7 | α = .97 | α = .98 |
| AWE-S | Time | 5 | 1-7 | α = .93 | α = .92 |
| AWE-S | Self-Diminishment | 5 | 1-7 | α = .93 | α = .93 |
| AWE-S | Connectedness | 5 | 1-7 | α = .94 | α = .92 |
| AWE-S | Vastness | 5 | 1-7 | α = .94 | α = .94 |
| AWE-S | Physiological | 5 | 1-7 | α = .90 | α = .91 |
| AWE-S | Accommodation | 5 | 1-7 | α = .86 | α = .90 |
| Intentional Pattern Perception | - | 10 | 1-11 | α = .81 | α = .82 |
| Supernatural Belief | - | 8 | 1-10 | α = .95 | α = .95 |
| Supernatural Control | - | 3 | 1-10 | α = .89 | α = .89 |
| Need for Cognitive Closure | - | 15 | 1-6 | α = .91 | α = .90 |
| Intolerance for Ambiguity | - | 3 | 1-6 | α = .85 | α = .79 |
| Mind Perception | Total Scale | 48 | 0-6 | α = .88 | α = .92 |
| Mind Perception | Human | 6 | 0-6 | α = .91 | α = .90 |
| Mind Perception | Baby | 6 | 0-6 | α = .57^a^ | α = .52^b^ |
| Mind Perception | Dead Woman | 6 | 0-6 | α = .96 | α = .98 |
| Mind Perception | Dog | 6 | 0-6 | α = .71 | α = .66^b^ |
| Mind Perception | God | 6 | 0-6 | α = .92 | α = .91 |
| Mind Perception | Robot | 6 | 0-6 | α = .74 | α = .84 |
| Mind Perception | Superman | 6 | 0-6 | α = .96 | α = .95 |
| Mind Perception | Tree | 6 | 0-6 | α = .91 | α = .95 |
| Prosocial Tendencies Measure | Total Scale | 25 | 1-5 | α = .87 | α = .78 |
| Prosocial Tendencies Measure | Altruism | 6 | 1-5 | α = .83 | α = .87 |
| Prosocial Tendencies Measure | Anonymous | 5 | 1-5 | α = .88 | α = .84 |
| Prosocial Tendencies Measure | Public | 4 | 1-5 | α = .86 | α = .91 |
| Prosocial Tendencies Measure | Emotional | 5 | 1-5 | α = .89 | α = .84 |
| Prosocial Tendencies Measure | Dire | 3 | 1-5 | α = .86 | α = .77 |
| Prosocial Tendencies Measure | Complaint | 2 | 1-5 | α = .78 | α = .68 |
| Satisfaction with Life Scale | - | 5 | 1-7 | α = .93 | α = .92 |

*Note:.^a^ The Baby Subscale of the Mind Perception Scale was the only scale not found to be reliable in Study 1; ^b^ The Baby Subscale and Dog Subscale of the Mind Perception Scale were the only scales not found to be reliable in Study 2.*
