# Supplementary material for: A series of replication attempts of experimental awe-induction effects
Source: PLoS One. 2026 Sep 2;21(9):e0356728. doi: 10.1371/journal.pone.0356728 (PMC13537573; doi:10.1371/journal.pone.0356728)
Supplement: Supplementary file 2 [file pone.0356728.s002.docx]

**Supplemental Materials**

A Series of Replication Attempts of Experimental Awe-Induction Effects

**S2 Table**

*Reliability Analyses for Study 3*

| Scale | Subscale | No. of Items | Range | Reliability |
| --- | --- | --- | --- | --- |
| PANAS | Negative Affect | 6 | 1-11 | α = .76 |
| PANAS | Positive Affect | 4 | 1-11 | α = .89 |
| AWE-S | Total Scale | 30 | 1-7 | α = .94 |
| AWE-S | Time | 5 | 1-7 | α = .88 |
| AWE-S | Self-Diminishment | 5 | 1-7 | α = .92 |
| AWE-S | Connectedness | 5 | 1-7 | α = .90 |
| AWE-S | Vastness | 5 | 1-7 | α = .92 |
| AWE-S | Physiological | 5 | 1-7 | α = .84 |
| AWE-S | Accommodation | 5 | 1-7 | α = .71 |
| Presence | - | 11 | 1-11 | α = .72 |
| Moral Circle | - | 30 | 1-4 | α = .95 |
| Primals | Interconnectedness | 4 | 1-6 | α = .87 |
| Primals | Safe | 6 | 1-6 | α = .77 |
| Primals | Enticing | 7 | 1-6 | α = .69 ^a^ |
| Primals | Alive | 5 | 1-6 | α = .83 |
| Intentional Pattern Perception | - | 10 | 1-11 | α = .78 |
| Supernatural Belief | - | 8 | 1-10 | α = .91 |
| Satisfaction with Life Scale | - | 5 | 1-7 | α = .87 |

Note. ^a^ *The Enticing sub-scale of Primals was the only scale not found to be reliable.*
