# Supplementary material for: A series of replication attempts of experimental awe-induction effects
Source: PLoS One. 2026 Sep 2;21(9):e0356728. doi: 10.1371/journal.pone.0356728 (PMC13537573; doi:10.1371/journal.pone.0356728)
Supplement: Supplementary file 3 [file pone.0356728.s003.docx]

**Supplemental Materials**

A Series of Replication Attempts of Experimental Awe-Induction Effects

**S3 Table**

*Correlations between Awe Item, AWE-S, Small Self and Primary Outcome Variables*

| ***Study 1 - Outcomes*** | | Awe Item | Awe-S | Small Self |
| --- | --- | --- | --- | --- |
| Life Satisfaction |  | .188** | .163** | .089* |
| Supernatural Control |  | .289** | .376** | .254** |
| Belief in God |  | .265** | .347** | .240** |
| Supernatural Belief |  | .313** | .403** | .279** |
| Prosocial Behavior |  | .187** | .254** | .130** |
| Intentional Pattern Perception |  | .114** | .154** | .134** |
| Uncertainty Intolerance |  | .040 | .042 | .030 |
| Mind Perception |  | .186** | .301** | .242** |
|  |  |  |  |  |
| ***Study 2 - Outcomes*** |  |  |  |  |
| Life Satisfaction |  | .213** | .259** | .203** |
| Supernatural Control |  | .195** | .391** | .293** |
| Supernatural Belief |  | .227** | .419** | .327** |
| Prosocial Behavior |  | .171** | .255** | .169** |
| Intentional Pattern Perception |  | .199** | .274** | .227** |
| Uncertainty Intolerance |  | .047 | .192** | .188** |
| Mind Perception |  | .269** | .416** | .380** |
|  |  |  |  |  |
| ***Study 3 - Outcomes*** |  |  |  |  |
| Life Satisfaction |  | .038 | .336** | .319** |
| Supernatural Control |  | .244* | .105 | .250* |
| Moral Circle |  | .003 | .112 | .205 |
| Primals - Interconnected |  | .044 | .132 | .259* |
| Primals - Safe |  | -.022 | .142 | -.081 |
| Primals - Alive |  | .045 | .153 | .185 |
| Intentionality |  | .088 | .207* | .333* |
| Presence |  | .250* | .214* | .327* |
| Inclusion of Self In Other |  | -.195 | -.087 | -.086 |
|  |  |  |  |  |
| ***Study 4 - Outcomes*** |  |  |  |  |
| Supernatural Control |  | .178** | - | - |
| Belief in God |  | .139* | - | - |
|  |  |  |  |  |
| ***Study 5 - Outcomes*** |  |  |  |  |
| Ethical Decision Making |  | .071 | - | .008 |
|  |  |  |  |  |
| ***Study 6 - Outcomes*** |  |  |  |  |
| Momentary Life Satisfaction |  | .011 | - | - |
| Perceived Time Availability |  | .078 | - | - |
| Preference for Experiential Goods |  | .038 | - | - |

p<.05*; p<.01**; - = not measured
