# Supplementary material for: A series of replication attempts of experimental awe-induction effects
Source: PLoS One. 2026 Sep 2;21(9):e0356728. doi: 10.1371/journal.pone.0356728 (PMC13537573; doi:10.1371/journal.pone.0356728)
Supplement: Supplementary file 4 [file pone.0356728.s004.docx]

**Supplemental Materials**

A Series of Replication Attempts of Experimental Awe-Induction Effect

**S1 File**

**Study 1 – Findings Across Outcome Measures**

**Supernatural Belief.** No significant differences were observed across the awe (*M* = 4.75, *SD* = 2.74), amusement (*M* = 4.69, *SD* = 2.78), or neutral (*M* = 4.60, *SD* = 2.69) conditions. While there was a nominal increase in the awe condition, these differences were not found to be significant, [*F*(2, 624) = .161, *p* = .851, *η*^2^ = .00]. Furthermore, no significant difference in supernatural belief was found when comparing the awe condition (*M* = 4.75, *SD* = 2.74) to only the neutral condition (*M* = 4.60, *SD* = 2.69), [*t*(445) = .570, *p* = .569].

There were no significant differences in belief supernatural control reported between the awe (*M* = 4.93, *SD* = 2.92), amusement (*M* = 4.94, *SD* = 2.99), or neutral (*M* = 4.76, *SD* = 2.92) conditions, [*F*(2, 624) = .269, *p* = .764, *η*^2^ = .00]. Furthermore, no significant difference in belief in supernatural control was found when comparing the awe condition (*M* = 4.93, *SD* = 2.92) to only the neutral condition (*M* = 4.76, *SD* = 2.92), [*t*(445) = .636, *p* = .525].

**Intentional Pattern Perception.** No significant differences in ratings of intentional pattern perception between the awe condition (*M* = 5.39, *SD* = 1.77), the amusement condition (*M =* 5.42, *SD* = 1.85), and the neutral condition (*M* = 5.54, *SD* = 1.64), [*F*(2, 624) = .468, *p* = .626, *η*^2^ = .00]. Furthermore, no significant difference was found when comparing the awe condition (*M =* 5.42, *SD* = 1.85) to only the neutral condition (*M* = 5.54, *SD* = 1.64), [*t*(445) = -.943, *p* = .346].

**Uncertainty Tolerance.** No significant differences were found in need for cognitive closure between the awe condition (*M* = 3.88, *SD* = .92), the amusement condition (*M =* 3.91, *SD* = .94), and the neutral condition (*M* = 3.84, *SD* = .97), [*F*(2, 624) = .228, *p* = .796, *η*^2^ = .00]. Furthermore, no significant difference was found when comparing the awe condition (*M* = 3.88, *SD* = .92) to only the neutral condition (*M* = 3.84, *SD* = .97), [*t(*445) = .434, *p* = .664].

Additionally, no significant differences in intolerance for ambiguity were found between awe condition (*M* = 3.87, *SD* = 1.25), the amusement condition (*M =* 4.03, *SD* = 1.26), and the neutral condition (*M* = 3.86, *SD* = 1.32), [*F*(2, 624) = 1.007, *p* = .366, *η*^2^ = .00]. Furthermore, when comparing the awe condition (*M* = 3.87, *SD* = 1.25) only to the neutral condition (*M* = 3.86, *SD* = 1.32) differences in intolerance for ambiguity were also not seen, [*t(*445) = .002, *p* = .998].

**Prosocial Behavior.** No significant differences in prosocial behavior were found between the awe (*M* = 3.24, *SD* = .58), amusement (*M* = 3.24, *SD* = .60), or neutral (*M* = 3.21, *SD* = .59) conditions, [*F*(2, 624) = .135, *p* = .874, *η*^2^ = .00]. Additionally, no differences in prosocial behavior were found between the awe (*M* = 3.24, *SD* = .58) and neutral (*M* = 3.21, *SD* = .59) conditions, [*t(*445) = .421, *p* = .674].

**Mind Perception.** No significant differences were found between the awe (*M* = 2.76, *SD* = .61), amusement (*M* = 2.71, *SD* = .75), and neutral conditions (*M* = 2.70, *SD* = .66), [*F*(2,624) = .876, *p* = .417, *η*^2^ = .00]. Furthermore, no significant difference was found when comparing the awe condition (*M* = 2.76, *SD* = .61) to only the neutral condition (*M* = 2.70, *SD* = .66), [*t*(445) = 1.392, *p* = .165].

**Satisfaction with Life.** There was a marginally significant relationship between awe and Satisfaction with Life. When comparing the awe condition (*M* = 4.65, *SD* = 1.53) to only the neutral condition (*M =* 4.35, *SD* = 1.73), a significant difference in life satisfaction was found, [*t(*445) = 1.973, *p* = .049]. This significant effect for life satisfaction did not exist, however, between the amusement (*M* = 4.42, *SD* = 1.61) and neutral (*M =* 4.35, *SD* = 1.73) conditions, [*t(*403) = .445, *p* = .657]. Significant differences for life satisfaction also did not exist between the awe and amusement (*M* = 4.42, *SD* = 1.61) conditions, [*t(*369) = 1.461, *p* = .145]. Furthermore, there were no significant differences for life satisfaction in an ANOVA of awe (*M* = 4.65, *SD* = 1.53), amusement (*M* = 4.42, *SD* = 1.61) and neutral (*M =* 4.35, *SD* = 1.73), [*F*(2,624) = 2.102, *p* = .123, *η*^2^ = .00].
