# Supplementary material for: A series of replication attempts of experimental awe-induction effects
Source: PLoS One. 2026 Sep 2;21(9):e0356728. doi: 10.1371/journal.pone.0356728 (PMC13537573; doi:10.1371/journal.pone.0356728)
Supplement: Supplementary file 5 [file pone.0356728.s005.docx]

**Supplemental Materials**

A Series of Replication Attempts of Experimental Awe-Induction Effects

**S2 File**

**Study 2 – Findings Across Outcome Measures**

**Supernatural Belief.** No significant differences were observed across the awe (*M* = 5.30, *SD* = 2.85), general positivity (*M* = 5.32, *SD* = 2.78), or neutral (*M* = 5.55, *SD* = 2.62) conditions, [*F*(2, 569) = .510, *p* = .601, *η*^2^ = 00]. Furthermore, no significant difference was found when comparing the awe condition (*M* = 5.30, *SD* = 2.85) to only the neutral condition (*M* = 5.55, *SD* = 2.62), [*t*(370.139) = .900, *p* = .368].

Additionally, no significant difference in belief supernatural control reported between the awe (*M* = 5.49, *SD* = 2.99), general positivity (*M* = 5.62, *SD* = 2.93), or neutral (*M* = 5.79, *SD* = 2.81) conditions, [*F*(2, 569) = .510, *p* = .601, *η*^2^ = .00]. Furthermore, no significant difference in belief in supernatural control was found when comparing the awe condition awe (*M* = 5.49, *SD* = 2.99) to only the neutral condition (*M* = 5.79, *SD* = 2.81), [*t*(379) = 1.007, *p* = .314].

**Intentional Pattern Perception.** No significant differences in ratings of intentional pattern perception between the awe condition (*M* = 5.98, *SD* = 1.57), the general positivity condition (*M =* 6.18, *SD* = 1.82), and the neutral condition (*M* = 5.79, *SD* = 2.03), [*F*(2,569) = 2.197, *p* = .112, *η*^2^ = .01]. Furthermore, no significant difference was found when comparing the awe condition (*M* = 5.98, *SD* = 1.57) to only the neutral condition neutral condition (*M* = 5.79, *SD* = 2.03), [*t(*366.639) = -1.052, *p* = .293].

**Uncertainty Tolerance.** No significant differences were found in need for cognitive closure between the awe condition (*M* = 4.06, *SD* = .94), the general positivity condition (*M =* 4.08, *SD* = .90), and the neutral condition (*M* = 4.16, *SD* = .06), [*F*(2,569) = .696, *p* = .499, *η*^2^ = 00]. Furthermore, no significant difference was found when comparing the awe condition (*M* = 4.06, *SD* = .94) to only the neutral condition (*M* = 4.08, *SD* = .90), [*t(*379) = 1.108, *p* = .269].

Additionally, no significant differences in intolerance for ambiguity were found between awe condition (*M* = 4.03, *SD* = 1.27), the general positivity condition (*M =* 4.08, *SD* = 1.24), and the neutral condition (*M* = 4.13, *SD* = 1.10), [*F*(2, 559) = .325, *p* = .772, *η*^2^ = .00]. Furthermore, when comparing the awe condition (*M* = 4.03, *SD* = 1.27) only to the neutral condition (*M* = 4.13, *SD* = 1.10) differences in intolerance for ambiguity were also not seen, [*t(*379) = .819, *p* = 413].

**Prosocial Behavior.** No significant differences in prosocial behavior between the awe (*M* = 3.33, *SD* = .49), general positivity (*M* = 3.32 , *SD* = .46), or neutral (*M* = 3.29, *SD* = .49) conditions were observed, [*F*(2, 568) = .312, *p* = .732, *η*^2^ = .00]. Furthermore, no significant difference was found when comparing the awe (*M* = 3.33, *SD* = .49) condition to only the neutral condition (*M* = 3.29, *SD* = .49), [*t(*378) = -.744, *p* = .458].

**Mind Perception.** No significant differences were found between the awe (*M* = 3.93, *SD* = .86), general positivity (*M* = 4.02 , *SD* = .89), and neutral conditions (*M* = 3.98, *SD* = .83), [*F*(2,569) = .491, *p* = .612, *η*^2^ = .00]. Furthermore, no significant difference was found when comparing the awe condition (*M* = 3.93, *SD* = .86) to only the neutral condition (*M* = 3.98, *SD* = .83), [*t(*379) = .617, *p* = .537, n^2^ = .00].

**Satisfaction with Life.** No significant differences were found in an ANOVA between awe (*M* = 4.53, *SD* = 1.57), general positivity (*M* = 4.83, *SD* = 1.47) and neutral (*M =* 4.53, *SD* = 1.58), [*F*(2,568) = 1.753, *p* = .174, *η*^2^ = .00]. Furthermore, no significant differences were found when comparing the awe condition (*M* = 4.53, *SD* = 1.57) to only the neutral condition (*M =* 4.63, *SD* = 1.58), [*t(*378) = .678, *p* = .498].
