# Supplementary material for: A series of replication attempts of experimental awe-induction effects
Source: PLoS One. 2026 Sep 2;21(9):e0356728. doi: 10.1371/journal.pone.0356728 (PMC13537573; doi:10.1371/journal.pone.0356728)
Supplement: Supplementary file 6 [file pone.0356728.s006.docx]

**Supplemental Materials**

A Series of Replication Attempts of Experimental Awe-Induction Effects

**S3 File**

**Study 3 – Findings Across Outcome Measures**

**Presence**. No significant differences were observed across the experimental (M=3.94, SD=1.28), active control (M=4.05, SD=1.20), or neutral control (M=4.62, SD=1.10) [*F*(2, 88) = 2.687, *p* = .074].

**Moral Circle**. No significant differences were observed across the experimental (M=42.38, SD=14.66), active control (M=44.30, SD=14.55), or neutral control (M=42.67, SD=14.38) [*F*(2, 88) = .156, *p* = .856].

**Primals**. For Interconnectedness, no significant differences were observed across the experimental (M=4.07, SD=.94), active control (M=3.90, SD=1.01), or neutral control (M=3.68, SD= .93) [*F*(2, 88) = 1.281, *p* = .283]. For Alive, no significant differences were observed across the experimental (M=3.68, SD=1.03), active control (M=3.56, SD=1.26), or neutral control (M=3.95, SD=.94) [*F*(2, 88) = .938, *p* = .395]. For Enticing, no significant differences were observed across the experimental (M=4.78, SD=.63), active control (M=4.62, SD=.83), or neutral control (M=4.77, SD=.58) [*F*(2, 485) = .938, *p* = .618]. For Safe, no significant differences were observed across the experimental (M=3.51, SD=.75), active control (M=3.41, SD=.76), or neutral control (M=3.64, SD=.70) [*F*(2, 485) = .659, *p* = .520].

**Intentionality**. No significant differences were observed across the experimental (M=5.17, SD=1.46), active control (M=5.35, SD=1.44), or neutral control (M=5.33, SD=2.03) [*F*(2, 88) = .111, *p* = .895].

**Supernatural Belief**. No significant differences were observed across the experimental (M=5.05, SD=2.21), active control (M=4.70, SD=2.49), or neutral control (M=4.98, SD=1.92) [*F*(2, 88) = .216, *p* = .806].

**Inclusion of Other in Self**. No significant differences were observed across the experimental (M=5.64, SD=2.22), active control (M=5.69, SD=1.95), or neutral control (M=5.96, SD=1.97) [*F*(2, 88) = .207, *p* = .813].

**Life Satisfaction**. No significant differences were observed across the experimental (M=4.92, SD=1.31), active control (M=4.65, SD=1.63), or neutral control (M=5.19, SD=1.38) [*F*(2, 88) = .966, *p* = .385].
