# Supplementary material for: A series of replication attempts of experimental awe-induction effects
Source: PLoS One. 2026 Sep 2;21(9):e0356728. doi: 10.1371/journal.pone.0356728 (PMC13537573; doi:10.1371/journal.pone.0356728)
Supplement: Supplementary file 7 [file pone.0356728.s007.docx]

**Supplemental Materials**

A Series of Replication Attempts of Experimental Awe-Induction Effects

**S4 File**

**Study 6 - Prompts**

*[Awe:] Imagine you’re getting ready to go on a trip to Europe. Although you’ve seen parts of Europe in photos and on television, you know that seeing things in person will be a completely different experience. You’re particularly excited to begin the trip in one of the most inspiring capitals of the continent—the magnificent city of Paris. 

As soon as you arrive in Paris, you’re overwhelmed by the grandeur and beauty of the historic city. The sights, smells, and sounds are like nothing you have ever experienced. Everywhere you look there is something new to capture your imagination. Scanning the view from left to right, you’re surrounded by beautiful buildings. Famous museums and churches beckon for you to absorb the stories of their rich past, while centuries-old hotels and city buildings exude majesty and history. As you pass by them, you’re amazed by the elaborate architectural designs and the ornate details. Between two of the buildings, you catch a glimpse of the Eiffel tower in the distance. Seeing it for the first time in person, your eyes widen and your senses feel wide awake. Although it looks small from where you are, the incredible height of the tower becomes clear as you walk toward it. 

Standing a block from the tower, you’re overwhelmed by the sheer size and grandeur of the structure. The intricately woven beams of steel rise high from the ground, and you feel completely dwarfed standing next to it. You look up, but you can’t even see the top. The magnitude of the tower is enormous and it feels even more amazing being there in person than you could have ever imagined. The metal beams rising from the ground are larger than the biggest tree trunks you’ve ever seen. You touch them: As your hands come in contact with the cold metal, you feel the presence of something greater than yourself, not just physically, but in human history. You can’t believe that something so tremendous was built by man. 

You take the elevator to the top. During the ride you can’t help but think back to the first time you saw the Grand Canyon—that moment when everything around you stops as you try to comprehend what’s in front of you. Finally, the elevator doors begin to open, and there it is—Paris all around you. As you take in the overwhelming sight, your mouth opens and you catch your breath. The famed City of Lights stretches for miles in all directions around you, yet from this vantage point the hustle and bustle below cease to exist. As your body is enveloped by a strong feeling of wonder, you scan the enormous panorama and try to take in everything that’s in front of you. You lose yourself in the beauty of the sight.*

*[Neutral:] Imagine you're getting ready to go on a trip. Although you've seen your destination in photos and on television, you know that seeing things in person will be a different experience. You're also thinking about how you've planned to begin the trip in a city that reminds you a lot of the place where you currently live. 

When you arrive at your destination, you step out into the street and take a look around. The sights, smells, and sounds are all very familiar to you. Everywhere you look, there is something that reminds you of your prior experiences. Scanning the view from left to right, you're surrounded by buildings and winding streets. A museum and a church are located at the end of the block, while several hotels and city buildings line the sides of the street. As you pass by them, you note the familiar colors and architectural designs and details. Between two of the buildings, you catch a glimpse of a tower in the distance. You decide that you want to take a closer look at the tower. After checking for passing cars, you cross the street and begin walking toward the tower. 

Standing a block from the tower, you can more clearly observe the shape of the structure and the materials that it was constructed from. The woven beams of steel rise from the ground, and decorative flowers and other foliage surround the base of the beams. You reach the tower and stand beside it, looking up towards the top. The metal beams forming the foundation of the tower glint in the sunlight. You touch them. Your hands come into contact with the cold metal, and you rap your knuckles against the beam. You listen as the sound echoes and can feel the vibrations reverberate through the steel. 

You walk to the entrance of the tower and press the button for the elevator. When the elevator doors open, the other passengers shuffle to the side to make room, and you step inside. As you take the elevator to the top, you can hear the quiet music that is being piped through the speakers. Finally, the elevator doors begin to open and you step out onto the viewing platform. You look down from the tower. You can see a few trees and many of the buildings that you passed on your walk to the tower. A gentle breeze brushes against your face, and when you listen carefully, you can hear the muffled sounds of the city below.*
