# Supplementary material for: A series of replication attempts of experimental awe-induction effects
Source: PLoS One. 2026 Sep 2;21(9):e0356728. doi: 10.1371/journal.pone.0356728 (PMC13537573; doi:10.1371/journal.pone.0356728)
Supplement: Supplementary file 8 [file pone.0356728.s009.docx]

Reviewer #1: This study examined the replicability of the cognitive and behavioral effects of awe—namely increased agency detection (Valdesolo & Graham, 2014), increased prosocial behavior (Piff et al., 2015), and enhanced well-being (Rudd et al., 2012)—using multiple awe-induction methods (videos, texts, and virtual reality) across both online and laboratory settings. Overall, the findings suggest that these effects were not clearly replicated under the conditions of the present research.

At the same time, the study does not fully qualify as a strict direct replication due to differences in participant pools and research platforms. Therefore, the tone of the claim that the effects “failed to replicate” may be somewhat too strong, and a more cautious interpretation may be warranted. That said, the authors’ effort to test replicability across diverse methods is commendable, and the fact that these effects did not emerge consistently across such varied conditions provides important insights into the robustness of the original findings. Below I provide specific comments.

1. Clarification of methodological differences from the original studies: Although the present work attempts to replicate prior findings using multiple approaches, many of the studies do not closely match the original studies in terms of participants, platforms, and procedures. For example, Study 4 is described as a direct replication of Valdesolo & Graham (2014) Study 1; however, the original study recruited participants on a university campus and conducted the experiment on “iPads,” whereas the present Study 4 was conducted online using Prolific. It would therefore strengthen the manuscript to more clearly describe the participant characteristics, research environments, platforms, and measured variables in the original studies, and to explicitly specify how Studies 1–6 differ from them. This would support a more cautious and balanced interpretation of the findings.

**We agree that we could better clarify which studies were strict, direct replications and which were very similar but not exact. We have made several changes to the manuscript to clarify this point:**

- **First, we have added a Table which describes the original studies (pg 5) and which ones we attempted to replicate (shaded and in bold).**
- **Second, we have clarified our use of the phrases "direct" for exact replications and "systematic" for merely similar replications (pg 7).**
- **Finally, we have included a stronger rationale for delivering Study 4 using an online sample versus a campus sample. (pg. x) Each of these changes are provided below for ease of reference.**

**Table 1**

**Overview of Original Studies**

| **Agency Detection - Valdesolo & Graham (2014)** | | |
| --- | --- | --- |
| ***Study (N)*** | ***Awe Induction Method*** | ***Outcomes*** |
| **Study 1***  **(N=120)** | **Videos:**   - **awe (panoramic scenes of natural beauty such as mountains)** - **amusement (comedic scenes from nature such as monkeys playing)** - **neutral (an interview)** | **Beliefs in supernatural control increased in the awe condition** |
| Study 2  (N=81) | Same videos as Study 1 | Uncertainty intolerance mediated the effect of awe on belief in supernatural control |
| Study 3  (N= 120) | Same videos as Study 1 | Agency detection mediates the effect of the manipulation condition on awe |
| Study 4  (N=76) | Neutral or Awe Videos from Study 1 | Uncertainty intolerance mediated the relationship between awe and agency detection |
| Study 5  (N=112) | Videos:   - awe - commercial with natural scenes) - amusement - commercial with happy scenes) | awe increased supernatural beliefs |
| **Prosociality - Piff et al. (2015)** | | |
| ***Study (N)*** | ***Awe Induction Method*** | ***Outcomes*** |
| Study 1 N=1519 | (no induction) | Dispositional awe is associated with dispositional prosocial tendencies |
| **Study 2***  **(N=75)** | **Writing Recall Task:**   - **awe - memory of an awe experience** - **Pride - memory of a pride experience** - **Neutral - memory of a recent event of any kind** | **Ethical decision making increased only in the awe condition** |
| Study 3  (N=264) | Videos:   - Same as Valdesolo & Graham's (2014) awe and amusement videos - Neutral - wooden countertop | Increased prosocial values for the awe condition |
| Study 4 | Videos   - awe - scientific footage of water droplets colliding at high magnification in slow motion, threatening natural scenes (e.g., tornado) - neutral - wooden countertop | Greater generosity in the dictator game for the awe condition |
| Study 5 | In Vivo   - awe - look at tall trees - neutral - look at campus building | Increased helping behavior (picking up pens) and reduced entitlement in the awe condition |
| **Life Satisfaction - Rudd et al., (2012)** | | |
| ***Study (N)*** | ***Awe Induction Method*** | ***Outcomes*** |
| Study 1 (N=63) | Videos   - awe - commercial with natural scenes - control - commercial with happy scenes | More perceived time availability in the awe condition |
| Study 2  (N=86) | Writing Recall Task   - awe - memory of an awe experience - control - memory of a happy experience | Those in awe condition were more willing to donate time and money |
| **Study 3***  **(N=105)** | **Story Visualization**   - **awe - read a story about climbing to the top of the Eiffel tower to look out at Paris** - **control - read a story about an unexceptional tower and landscape** | **Those in the awe condition reported more momentary life satisfaction, as well as preference for experiential vs. material goods** |

*Note*: Bolded and highlighted denote the specific original studies that systematic and direct replications were based on.

***"****In a series of 6 distinct studies, we attempt to replicate these outcomes associated with awe using two types of replications conceptual vs. direct (33). We use the term systematic replication to mean repeating studies conceptually, with intentional changes to the methodology to see whether the effects hold under new conditions. On the other hand, a direct replication involves repeating studies conceptually and methodologically as closely as possible to see if the same result can be reproduced. In the first series of studies in this paper, we conduct systematic replications, which were designed to expand upon the work of previous authors by including novel hypotheses and multiple outcomes (see Table 1). In the case of direct replications, we consulted with and received materials from the original lead authors to conduct high-fidelity versions of their original work. For all replications the same analytic techniques were used to examine differences between three (ANOVAs) or two (t-tests) between conditions, as well as the same partial eta squared (η_p_²) effect size measure to allow for comparison across studies." (PG 7)*

*"Study 4 in our study was a direct replication of Valdesolo and Graham's (2014; Study 1) video manipulation to elicit awe, amusement, or no emotion (neutral). Several authors (M.G., D.B.Y.) have already conducted an exact direct replication using a college campus sample as the authors did in the original study, similarly finding that the awe manipulation was successful, but outcomes did not replicate (Yaden et al., in press). To replicate this study with a bigger sample, an online sample was chosen with the acknowledgment that though this is a slight methodological change, in the original study the students on campus were still given an iPad to view the videos online. Given that this was the only change, we classify and report this study as a direct replication as the methodology was followed as closely as possible (see definition on page 7).*

*Like the original study, we expected the experimental awe condition would result in changes to supernatural control and belief in God relative to the active and passive control conditions." (PG 32)*

2. Strength of the awe manipulation (manipulation check): It would be important to report how the effect sizes of the subjective awe measures compare with those reported in the original studies. Were they significantly different? If the awe induction was relatively weaker in the present research, this may help explain the pattern of results.

**Thank you for this helpful suggestion. We have now created a Table to provide a reader friendly comparison of the effect sizes from the original studies that we replicate, and those studies reported int he paper on page 3. The table is reproduced below:**

**Table 3**

**Awe Manipulation Effect Size Comparison to Original Studies**

| ***Original Study*** | ***Replication*** |
| --- | --- |
| *Valdesolo & Graham (2014)*   - *Study 1 -* ηp²*=.47* (large) | *Systematic*   - *Study 1 -* ηp²*=.25* (large)   *Direct*   - *Study 4 -* ηp²*=.30* (large) |
| *Piff et al. (2014)*   - *Study 2 - NR* | *Systematic*   - *Study 2 -* ηp²*=.15* (large)   *Direct*   - *Study 5 -* ηp²*=.31* (large) |
| *Rudd et al. (2012)*   - *Study 3 -* ηp²*=.04* (small) | *Systematic*   - *Study 3 -* ηp²*= .09* (medium)   *Direct*   - *Study 6 - η_p_²=.01^NS^* (small) |

*Note*. NR = Not reported. NS = Nonsignificant effect. Effect magnitude labels correspond to conventional benchmarks for partial eta squared (*ηp²*): small = .01, medium = .06, large = .14 (37,38),

3. Additional analyses linking awe-related measures and outcomes: Relatedly, I encourage the authors to examine correlations between Awe, AWE-S, Small Self, and all outcome variables. It is possible that participants who experienced stronger awe may have shown effects more consistent with the original findings.

**As this is a series of replications, we did not want to break with the original studies which do not conduct such analyses. However, given that the small-self has been identified as a feature or facet of awe in the time since these studies were published, we do agree that examining the correlation between small-self and outcomes is warranted. We now provide a correlation matrix in the supplement, and have produced it here below:**

**Table 3**

*Correlations between Awe Item, AWE-S, Small Self and Primary Outcome Variables*

| ***Study 1 - Outcomes*** | | Awe Item | Awe-S | Small Self |
| --- | --- | --- | --- | --- |
| Life Satisfaction |  | .188** | .163** | .089* |
| Supernatural Control |  | .289** | .376** | .254** |
| Belief in God |  | .265** | .347** | .240** |
| Supernatural Belief |  | .313** | .403** | .279** |
| Prosocial Behavior |  | .187** | .254** | .130** |
| Intentional Pattern Perception |  | .114** | .154** | .134** |
| Uncertainty Intolerance |  | .040 | .042 | .030 |
| Mind Perception |  | .186** | .301** | .242** |
|  |  |  |  |  |
| ***Study 2 - Outcomes*** |  |  |  |  |
| Life Satisfaction |  | .213** | .259** | .203** |
| Supernatural Control |  | .195** | .391** | .293** |
| Supernatural Belief |  | .227** | .419** | .327** |
| Prosocial Behavior |  | .171** | .255** | .169** |
| Intentional Pattern Perception |  | .199** | .274** | .227** |
| Uncertainty Intolerance |  | .047 | .192** | .188** |
| Mind Perception |  | .269** | .416** | .380** |
|  |  |  |  |  |
| ***Study 3 - Outcomes*** |  |  |  |  |
| Life Satisfaction |  | .038 | .336** | .319** |
| Supernatural Control |  | .244* | .105 | .250* |
| Moral Circle |  | .003 | .112 | .205 |
| Primals - Interconnected |  | .044 | .132 | .259* |
| Primals - Safe |  | -.022 | .142 | -.081 |
| Primals - Alive |  | .045 | .153 | .185 |
| Intentionality |  | .088 | .207* | .333* |
| Presence |  | .250* | .214* | .327* |
| Inclusion of Self In Other |  | -.195 | -.087 | -.086 |
|  |  |  |  |  |
| ***Study 4 - Outcomes*** |  |  |  |  |
| Supernatural Control |  | .178** | - | - |
| Belief in God |  | .139* | - | - |
|  |  |  |  |  |
| ***Study 5 - Outcomes*** |  |  |  |  |
| Ethical Decision Making |  | .071 | - | .008 |
|  |  |  |  |  |
| ***Study 6 - Outcomes*** |  |  |  |  |
| Momentary Life Satisfaction |  | .011 | - | - |
| Perceived Time Availability |  | .078 | - | - |
| Preference for Experiential Goods |  | .038 | - | - |

p<.05*; p<.01**; - = not measured

4. Tone of the conclusions: Given the methodological differences noted above and the potential value of additional analyses, the conclusion that the effects did not replicate may be too strong. After fully discussing these issues in the limitations section, it may be more appropriate to frame the findings as showing that the effects were “not replicated under the present conditions.”

**We have now elaborated on the difference between systematic and direct replications (see Comment 1) and have accordingly reframed our discussion of the findings considering the reviewer’s comments. For systematic (non-direct) replications we have added language to indicate that the effects were not replicated under the present conditions. As an example:**

*"Under the present conditions, we did not replicate the finding reported in Valdesolo and Graham (2014), Piff and colleagues (2015); and Rudd and colleagues (2012), though it should be noted that they used a few different forms of emotion inductions, especially video but also writing exercises." (PG 17)*

Reviewer #2: The authors have presented a fascinating and much-needed piece of work, taking the topical theme of awe and examining its effects on a wide range of positive outcomes. At a time when it is commonplace to discuss high-level positive experiences such as well-being and prosociality, it is commendable that the researchers have sought to test the validity of these claims empirically. Overall, this is an important and promising piece of work that has the potential to make a valuable contribution to the field. However, there are a number of points on which I seek clarification, outlined below. I hope that addressing the comments could further strengthen the manuscript.

In the literature review, the authors cite influential works in the field, such as Keltner and Haidt. However, there are a number of recent works that could help further contextualise the present work. For example, considering that the authors characterise awe as a complex emotional experience that includes both fear and positivity, and that awe relates to the sublime, the authors may consider the following works that support that.

Gordon, A. M., Stellar, J. E., Anderson, C. L., McNeil, G. D., Loew, D., & Keltner, D. (2017). The dark side of the sublime: Distinguishing a threat-based variant of awe. Journal of Personality and Social Psychology, 113(2), 310–328. <https://doi.org/10.1037/pspp0000120>

Hur, Y.-J., Gerger, G., Leder, H., & McManus, I. C. (2020). Facing the sublime: Physiological correlates of the relationship between fear and the sublime. Psychology of Aesthetics, Creativity, and the Arts, 14(3), 253–263. <https://doi.org/10.1037/aca0000204>

It should be noted that a work that connects the sublime with awe is the Clewis et al. (2022) work, which the authors have included in the reference section, but which I cannot see in the main text.

Considering that the authors view awe as being triggered through nature-related vast objects, it may be worth considering the following empirical works, which find a link between objective/subjective vastness and awe/sublime:

Ishizu, T., & Zeki, S. (2014). A neurobiological enquiry into the origins of our experience of the sublime and beautiful. Frontiers in Human Neuroscience, 8, 891. <https://doi.org/10.3389/fnhum.2014.00891>

Hur, Y.-J., Hallam-Evans, C., Garfen, Y., Baiza, A., Backhouse Spriggs, T., Mircea, M.-T., Nagy, O., Pye, E., & McManus, I. C. (2024). Differentiating the visual aesthetics of the sublime and the beautiful: Selective effects of stimulus size, height, and color on sublimity and beauty ratings in photographs. Psychology of Aesthetics, Creativity, and the Arts, 18(6), 904–920. <https://doi.org/10.1037/aca0000480>

**We have added content to the introduction which includes the citations you have suggested:**

*"The notion of awe can be traced back to the concept of “the sublime” in aesthetics, as articulated by Edmund Burke, Immanuel Kant, and others (3). Contemporary scholarship has continued to examine the link between awe and the sublime, suggesting that these experiences share core features including perceptual vastness, cognitive accommodation, and complex states of simultaneous positive and negative emotion, including elements of fear (3–5). Empirical work further supports the importance of subjective and objective features (e.g., height, scale, magnitude) in creating a sense of vastness in both sublime and awe experiences (6,7). Awe as a distinct psychological construct was first systematically outlined in the seminal paper “Approaching awe, a moral, spiritual, and aesthetic emotion” by Keltner and Haidt. They articulate two necessary qualities of awe: (1) perceptual vastness and (2) a need for accommodation. First, awe involves contact with a perceptually vast stimulus and second, a need for accommodation on behalf of the experiencer, which entails the need for a cognitive shift in one’s understanding of these stimuli (2)." (Pgs. 3-4).*

Overall, I found the citation system difficult to follow. The in-text citations appear to draw on two different systems; under APA style, citations should identify authors rather than use numeric references. Also, because some citations are presented numerically, it is not possible to reliably match the in-text citations to the reference list.

**Thank you for catching this. As PLos One requires Vancouver for submissions, the entire manuscript, including the reference list, has been converted to this citation style.**

Regarding the studies, whether systematic or direct replication studies, I appreciate that the same emotional induction technique and measures were used (p. 9). Please clarify if the same analytical methods were also used, as the analytical methods can often be linked with different outcomes.

**Thank you. We have clarified the difference between the systematic and direct replications and have added a sentence about the analytic technique (pg 7).**

***"****In a series of 6 distinct studies, we attempt to replicate these outcomes associated with awe using two types of replications: conceptual vs. direct (33). We use the term conceptual replication to mean repeating studies conceptually, with intentional changes to the methodology to see whether the effects hold under new conditions. On the other hand, a direct replication involves repeating studies conceptually and methodologically as closely as possible to see if the same result can be reproduced. In the first series of studies in this paper, we conduct systematic replications, which were designed to expand upon the work of previous authors by including novel hypotheses and multiple outcomes (see Table 1). In the case of direct replications, we consulted with and received materials from the original lead authors to conduct high-fidelity versions of their original work. For all direct replications the same analytic techniques were used to examine differences between three (ANOVAs) or two (t-tests) between conditions, as well as the same partial eta squared (η_p_²) effect size measure to allow for comparison across studies." (PG 7)*

I have noticed that whereas all the studies presented were run online, it may not always be the case that the original studies were run online. If the study context has changed, this should be mentioned and discussed in the paper (especially in light of the lack of replication of results).

**This is only the case for one of the three original studies we replicate. Valdesolo & Graham (2014) had college students watch the videos online using an iPad. So, there was an in-person element that we did not replicate here. We have clarified this in the manuscript on page 32:**

*"Study 4 in our study was a direct replication of Valdesolo and Graham's (2014; Study 1) video manipulation to elicit awe, amusement, or no emotion (neutral). Several authors (M.G., D.B.Y.) have already conducted an exact direct replication using a college campus sample as the authors did in the original study, finding that the awe manipulation was successful, but outcomes did not replicate the effects shown in the original study (Yaden et al., in press). To repeat this study with a bigger sample, an online sample was chosen with the acknowledgment that though this is a methodological change, in the original study the students on campus were still given an iPad to view the videos online. Given that this was the only change, we classify and report this study as a direct replication as the methodology was followed as closely as possible (see definition on pg. 7).*

*Like the original study, we expected the experimental awe condition would result in changes to supernatural control and belief in God relative to the active and passive control conditions." (Pg 32)*

The paper by Piff et al. examines prosocial values across a large number of studies. Please clarify the rationale for selecting only Study 2 from Piff et al. for replication. Although the authors state (p. 28) that only studies amenable to online replication were selected, it appears that several of the other studies reported by Piff et al. could also have been conducted online. Could the authors clarify why Study 2 was selected in the Piff paper?

**We were interested in the use of a writing recall task so that we could look at a diversity of induction techniques in addition to a diversity of outcomes (in other studies, we repeat studies from other publications using different induction methods such as videos). We consulted with Piff and they were able to provide us with the materials for Study 2, we have denoted this collaboration in the manuscript (see excerpt in previous comment).**

For all tables (and wherever inferential statistics are involved), please present effect sizes.

**We have added effect sizes throughout the manuscript.**

On page 28, the following sentence is not suitable as an introduction to the next set of studies, as it reveals the outcomes in advance: “Despite these changes, we continued to find the same pattern wherein awe was reported to be significantly induced, but no difference in outcome was statistically significant - except in one case where the awe manipulation failed (see Study 6)”

**Thank you, we have deleted this sentence from the manuscript.**

Certain sections of Study 6 (verbal prompts for experimental) would be more appropriate as supplementary material.

**We have moved these to the supplement - thank you for this suggestion.**

In studies of awe, experimental inductions often rely on a single stimulus, which may increase the potential for sample-specific variance. Could the authors please comment on this issue in the General Discussion section?

**We have added language to our discussion section to address this concern:**

*"Because many awe studies rely on a single stimulus induction, some sample-specific variability may remain. We attempted to mitigate this concern by employing different induction methods across studies." (Pg 48).*

The authors note (p. 44) that the magnitude of awe induction itself might account for the lack of observed changes in its psychological impact (e.g., well-being, prosociality). If so, it would be helpful for the authors to compare the effect size of awe induction in the present study with that reported in the original studies. It is possible that the original studies produced a larger effect, which could explain the observable changes in well-being, prosociality, and related outcomes.

**Thank you for this comment. We have created a Table to provide a reader friendly comparison of the effect sizes from the original studies that we replicate, and those studies reported in the paper on page 3. The table, showing that the replication study effect sizes of the emotion induction are broadly in line with the original studies, is reproduced below:**

**Table 3**

**Awe Manipulation Effect Size Comparison to Original Studies**

| ***Original Study*** | ***Replication*** |
| --- | --- |
| *Valdesolo & Graham (2014)*   - *Study 1 -* ηp²*=.47* (large) | *Systematic*   - *Study 1 -* ηp²*=.25* (large)   *Direct*   - *Study 4 -* ηp²*=.30* (large) |
| *Piff et al. (2014)*   - *Study 2 - NR* | *Systematic*   - *Study 2 -* ηp²*=.15* (large)   *Direct*   - *Study 5 -* ηp²*=.31* (large) |
| *Rudd et al. (2012)*   - *Study 3 -* ηp²*=.04* (small) | *Systematic*   - *Study 3 -* ηp²*= .09* (medium)   *Direct*   - *Study 6 - η_p_²=.01^NS^* (small) |

*Note*. NR = Not reported. NS = Nonsignificant effect. Effect magnitude labels correspond to conventional benchmarks for partial eta squared (*ηp²*): small = .01, medium = .06, large = .14 (37,38).

On pg. 43, the authors state that 31 out of 33 studies did not replicate the original studies. Please clarify how this sits with what was said in the abstract, that results were not replicated “except in one case” – some readers might find this confusing to understand.

**The language of this paragraph was confusing and so we have rewritten it as we did not conduct 33 studies, but rather looked at 33 outcomes.**

*"In the present series of studies, most of the findings were null with very few exceptions. Of 6 studies with 33 total outcomes, only a few were significant. In the systematic (non-direct) replications there was one significant outcome. In the direct replications there were two significant outcomes. These exceptions are described below. In Study 1, the video task was able to produce a significant effect on well-being when the awe condition was compared only to the neutral condition. This partially supported past research conducted by Rudd, Vohs, and Aaker (2012), which found that awe led to feelings of increased life satisfaction. Still, in the direct replication of this association (Study 6), this significant relationship went away. Additionally, in Study 2, the writing task was not able to reproduce this outcome of increased feelings of life satisfaction and awe. Similarly, in Study 4, a video task was able to produce significant differences in supernatural control when the awe condition was compared solely with the neutral condition. In a pre-registered direct replication (PDR; Yaden et al., under review), which used a similar sized sample (N=296), neither frequentist analyses nor Bayesian analyses replicated the significant effect of awe on supernatural control when looking only at the awe and neutral conditions. Finally, in Study 5, there was a statistically significant effect of awe small-self ratings, which replicated past research by Piff et al. (2014)." (pg. 47-48))*

**We have also corrected the abstract:**

**Abstract**

*"The self-transcendent emotion of awe has recently received research attention beyond many other emotions due to several intriguing outcomes reported in the experimental literature. Specifically, after inducing awe, Valdesolo and Graham (2014) found that agency detection is increased, Piff and colleagues (2015) found that prosocial behaviors are more likely, and Rudd and colleagues (2012) found that well-being is enhanced. This article tried to systematically replicate some of these effects in online and laboratory settings using video, writing, and virtual reality awe inductions. Results from these previous studies were generally not reproduced. To further investigate these failures to reproduce previously reported effects of awe, we conducted three direct replications with 2.5 times the original sample sizes, and past findings also were not reproduced. We discuss these findings in terms of research on emotion induction tools and provide recommendations for future research on awe."*

Given that the authors have conducted 33 studies with similar designs and aims, they might consider performing a meta-analysis (e.g., a random-effects meta-analysis with a forest plot) of their findings. This would help consolidate evidence that, across studies, (1) awe induction occurs, but (2) its impact on positive outcomes is negligible. By assessing the overall effect of the manipulation across multiple studies, such an analysis could also provide a valuable interpretation of the single study in which a replication effect was indeed observed.

**See above comment clarifying that 33 refers to outcomes from 6 studies, rather than 33 studies. Though we do agree that a separate, dedicated meta-analytic project that systematically aggregates a larger set of awe manipulation studies would be valuable, this project is outside of the scope of the present manuscript.**
