# Supplementary material for: A series of replication attempts of experimental awe-induction effects
Source: PLoS One. 2026 Sep 2;21(9):e0356728. doi: 10.1371/journal.pone.0356728 (PMC13537573; doi:10.1371/journal.pone.0356728)
Supplement: Supplementary file 9 [file pone.0356728.s010.docx]

**Editor Comments:**

In particular, please note that PLOS ONE considers psychology or behavioural research studies conducted using Amazon MTurk / Survey Monkey for publication. However, during the evaluation of your manuscript, we would be grateful if you could carefully address the limitations commonly associated with this type of study—namely, the **non‑naivety and trustworthiness of participants**. You should explicitly discuss how these issues were handled in your study design, data collection, and quality assurance procedures.

**Thank you, we have added language (reproduced below) to address the limitations of MTurk in the two studies in which we recruited participants via MTurk (Study 1 and Study 2). Note that the other online samples were collected via Prolific (Study 4-6) and Study 3 was an in-person laboratory study.**

*"Although data for two of the six studies were collected via Amazon Mechanical Turk, concerns regarding participant non-naivety and response trustworthiness were addressed through multiple quality assurance procedures (e.g., attention checks, eligibility screening, and exclusion of low-quality responses), although these concerns remain an inherent limitation of online convenience samples." (Pg. 9)*

Additionally, please consider whether you need to **validate the results using another non‑web‑based sample** (e.g., a laboratory or community sample). If you believe such validation is not necessary, please provide a clear and compelling justification in your revision.

**As above, note that we did conduct a laboratory study reported in this manuscript. We have also conducted an in-person laboratory study cited in the manuscript with the same results.**

**We do not believe that further validation using a non-web-based sample is necessary in this case. First, the pattern of results was highly consistent across methodologies and settings. Specifically, null findings were observed not only in large online samples but also in a laboratory-based virtual reality study (Study 3) and are consistent with independent in-person replication attempt we have published elsewhere. Taken together, these convergent findings suggest that the lack of replication is unlikely to reflect artifacts of online data collection. We include reference to the results of our in-person pre-registered direct replication of Valdesolo and Graham's Study. The language is reproduced below:**

*"Study 4 in our study was a designed as a direct replication of Valdesolo and Graham's (2014; Study 1) video manipulation to elicit awe, amusement, or no emotion (neutral). Several authors (M.G., D.B.Y., along an original author, Valdesolo) have previously conducted a pre-registered direct replication using an in-person college campus sample as the authors did in the original study, similarly finding that the awe manipulation was successful, but outcomes from the original study did not replicate (Yaden et al., in press). To increase statistical power and sample size, an online Prolific sample was chosen rather than recruiting participants from a college campus. We acknowledge that this represents a methodological deviation from the original study. However, the participants in our study viewed the exact same video stimuli that the original study participants viewed on iPads, and the measures, procedures, and analytic approach were otherwise matched to those used by the original authors. Consistent with our definition of direct replication (p.7), we classify this study as a direct replication while recognizing that the change may have influenced the findings." (Pg. 29)*

**Reviewer #1**

Thank you for the careful revisions. The manuscript has improved substantially, and my main concerns have largely been addressed. I have only a few remaining minor comments.

1. In the response letter, the authors state that they have clarified their use of the terms “direct” for exact replications and “systematic” for merely similar replications. However, this clarification does not appear to be fully reflected in the manuscript. In the manuscript, the distinction is still framed primarily as “conceptual” versus “direct” replication, and the relationship between “conceptual” and “systematic” replication remains unclear. Please ensure that these terms are used consistently and that the intended distinction is clearly reflected in the manuscript.

**Thank you for highlighting this inconsistency. In the response letter the use of the word systematic was an error. We have eliminated the word "systematic" throughout the manuscript and replaced it with "conceptual", which is terminology grounded in the research literature to describe these different approaches to replication. We have clarified this further in the body of the manuscript and included additional citations for this language.**

***"****In a series of 6 distinct studies, we attempt to replicate these outcomes associated with awe using two types of replications: conceptual vs. direct (33, 34, 35). The term "conceptual replication" is used throughout the literature to mean repeating studies conceptually, with intentional changes to the methodology to see whether the effects hold under new conditions. On the other hand, a direct replication involves repeating studies conceptually and methodologically as closely as is possible to see if the same result can be reproduced. In the first series of studies in this paper, we conduct conceptual replications, which were designed to expand upon the work of previous authors by including novel hypotheses and multiple outcomes (see Table 1). In the case of direct replications, we consulted with and received materials from the original lead authors to conduct high-fidelity versions of their original work as closely as possible. For all replications the same analytic techniques were used to examine differences between three (ANOVAs) or two (t-tests) conditions, as well as the same partial eta squared (η_p_²) effect size measure to allow for comparison across studies." (P. 7)*

2. For Study 4, if it is retained as a direct replication, please more explicitly acknowledge the methodological difference from the original study, namely the shift from a campus/iPad setting to an online Prolific sample. This difference should also be briefly noted in the limitations, and statements such as “this was the only change” may need to be phrased more cautiously.

*"Study 4 in our study was a designed as a direct replication of Valdesolo and Graham's (2014; Study 1) video manipulation to elicit awe, amusement, or no emotion (neutral). Several authors (M.G., D.B.Y., along an original author, Valdesolo) have previously conducted a pre-registered direct replication using an in-person college campus sample as the authors did in the original study, similarly finding that the awe manipulation was successful, but outcomes from the original study did not replicate (Yaden et al., in press). To increase statistical power and sample size, an online Prolific sample was chosen rather than recruiting participants from a college campus. We acknowledge that this represents a methodological deviation from the original study. However, the participants in our study viewed the exact same video stimuli that the original study participants viewed on iPads, and the measures, procedures, and analytic approach were otherwise matched to those used by the original authors. Consistent with our definition of direct replication (p.7), we classify this study as a direct replication while recognizing that the change may have influenced the findings." (P. 29)*

3. Please correct the inconsistent citation year for Piff et al., which appears as both 2014 and 2015. The manuscript should use the correct year consistently.

**Thank you, this has been updated throughout.**

4. The newly added exploratory correlations between self-reported awe, AWE-S, small self, and the outcome variables are informative. Although these analyses are correlational and exploratory, they suggest that, at the individual-difference level, participants who experienced stronger awe may have shown higher scores on some relevant outcomes. I recommend briefly mentioning these findings in the main text and discussing their interpretation cautiously in the Discussion, rather than leaving them only in the supplement.

**Thank you for raising this point. We have included language in the results and discussion section referencing the correlational analyses (reproduced below):**

*"Although the experimental manipulations generally did not produce significant between-group differences, exploratory analyses indicated that participants reporting greater subjective experiences of awe tended to report higher scores on several outcome measures (see Supplementary Table 3 for exploratory correlations for each of the six studies in this paper)." (P. 15)*

*"Although experimental manipulations largely failed to produce differences between conditions, exploratory correlations indicated that individuals who reported stronger experiences of awe tended to score higher on several outcomes. Though causal conclusions cannot be drawn, these analyses raise the possibility that the magnitude or intensity of the awe experience may be an important feature to examine in future research." (P. 43)*

Overall, I find the authors’ revisions responsive, and these remaining issues appear minor but important for clarity and balanced interpretation.

**Reviewer #2**

The authors have addressed the initial concerns, and I thank them for their corrections. Please see my minor comments below:

On page 4, the authors ought to delete the “4/13/2026 1:33:00 PM”, as I am almost certain this is a typing mistake. Full section as it currently stands: “Other facets of awe include alterations to time (e.g., the sense that time slows down or speeds up; 4/13/2026 1:33:00 PM, small self…"

**Thank you for catching this artifact of our reference manager use. This sentence has been corrected.**

The authors have ruled out the possibility of a meta-analysis in the present analysis, citing that it is outside of the scope of the present manuscript. Could the authors please further elaborate?

**We appreciate the suggestion and agree that a meta-analysis of the broader field would be valuable. However, in the context of this manuscript we do not believe that a meta-analysis of the six studies would be appropriate because the studies have different outcomes. These outcomes include the following: well-being, supernatural control, belief in God, prosociality, and small self. The studies include different outcomes and even different outcome measures similar concepts preventing a meaningful meta-analysis.**
