# Supplementary material for: A series of replication attempts of experimental awe-induction effects
Source: PLoS One. 2026 Sep 2;21(9):e0356728. doi: 10.1371/journal.pone.0356728 (PMC13537573; doi:10.1371/journal.pone.0356728)
Supplement: Peer review history

# Peer review: Decision Letter 0

**DOI:** 10.1371/journal.pone.0356728.r001

**Authors:** Haixia Wang

25 Feb 2026

Dear Dr. Graziosi,

Thank you for submitting your manuscript to PLOS ONE. After careful consideration, we feel that it has merit but does not fully meet PLOS ONE’s publication criteria as it currently stands. Therefore, we invite you to submit a revised version of the manuscript that addresses the points raised during the review process.

==============================

This manuscript attempts to replicate awe-induced effects and holds considerable value. At the same time, the author may need to discuss the similarities and differences between the experimental design and procedures and those of previous related studies, particularly direct replication studies. Additionally, it is suggested that the author moderately discuss the reasons for the failure to replicate the findings, whether due to the absence of an effect, its small magnitude, experimental manipulation, or other factors.

==============================

Please submit your revised manuscript by Apr 11 2026 11:59PM. If you will need more time than this to complete your revisions, please reply to this message or contact the journal office at plosone@plos.org. When you're ready to submit your revision, log on to https://www.editorialmanager.com/pone/ and select the 'Submissions Needing Revision' folder to locate your manuscript file.

- A letter that responds to each point raised by the academic editor and reviewer(s). You should upload this letter as a separate file labeled 'Response to Reviewers'.
- A marked-up copy of your manuscript that highlights changes made to the original version. You should upload this as a separate file labeled 'Revised Manuscript with Track Changes'.
- An unmarked version of your revised paper without tracked changes. You should upload this as a separate file labeled 'Manuscript'.

If you would like to make changes to your financial disclosure, please include your updated statement in your cover letter. Guidelines for resubmitting your figure files are available below the reviewer comments at the end of this letter.

If applicable, we recommend that you deposit your laboratory protocols in protocols.io to enhance the reproducibility of your results. Protocols.io assigns your protocol its own identifier (DOI) so that it can be cited independently in the future. For instructions see: https://journals.plos.org/plosone/s/submission-guidelines#loc-laboratory-protocols. Additionally, PLOS ONE offers an option for publishing peer-reviewed Lab Protocol articles, which describe protocols hosted on protocols.io. Read more information on sharing protocols at https://plos.org/protocols?utm_medium=editorial-email&utm_source=authorletters&utm_campaign=protocols.

We look forward to receiving your revised manuscript.

Kind regards,

Haixia Wang

Academic Editor

PLOS One

Journal Requirements:

When submitting your revision, we need you to address these additional requirements.

1. Please ensure that your manuscript meets PLOS ONE's style requirements, including those for file naming. The PLOS ONE style templates can be found at

https://journals.plos.org/plosone/s/file?id=wjVg/PLOSOne_formatting_sample_main_body.pdf and

https://journals.plos.org/plosone/s/file?id=ba62/PLOSOne_formatting_sample_title_authors_affiliations.pdf

2. Please provide additional details regarding participant consent. In the ethics statement in the Methods and online submission information, please ensure that you have specified (1) whether consent was informed and (2) what type you obtained (for instance, written or verbal, and if verbal, how it was documented and witnessed). If your study included minors, state whether you obtained consent from parents or guardians. If the need for consent was waived by the ethics committee, please include this information.

If you are reporting a retrospective study of medical records or archived samples, please ensure that you have discussed whether all data were fully anonymized before you accessed them and/or whether the IRB or ethics committee waived the requirement for informed consent. If patients provided informed written consent to have data from their medical records used in research, please include this information

3. We note you have included a table to which you do not refer in the text of your manuscript. Please ensure that you refer to Table 6 in your text; if accepted, production will need this reference to link the reader to the Table

4. If the reviewer comments include a recommendation to cite specific previously published works, please review and evaluate these publications to determine whether they are relevant and should be cited. There is no requirement to cite these works unless the editor has indicated otherwise.

[Note: HTML markup is below. Please do not edit.]

Reviewers' comments:

Reviewer's Responses to Questions

**Comments to the Author**

1. Is the manuscript technically sound, and do the data support the conclusions?

Reviewer #1: Partly

Reviewer #2: Yes

**********

2. Has the statistical analysis been performed appropriately and rigorously?

Reviewer #1: Yes

Reviewer #2: Yes

**********

3. Have the authors made all data underlying the findings in their manuscript fully available?

The PLOS Data policy

Reviewer #1: Yes

Reviewer #2: Yes

**********

4. Is the manuscript presented in an intelligible fashion and written in standard English?

Reviewer #1: Yes

Reviewer #2: Yes

**********

Reviewer #1: This study examined the replicability of the cognitive and behavioral effects of awe—namely increased agency detection (Valdesolo & Graham, 2014), increased prosocial behavior (Piff et al., 2015), and enhanced well-being (Rudd et al., 2012)—using multiple awe-induction methods (videos, texts, and virtual reality) across both online and laboratory settings. Overall, the findings suggest that these effects were not clearly replicated under the conditions of the present research.

At the same time, the study does not fully qualify as a strict direct replication due to differences in participant pools and research platforms. Therefore, the tone of the claim that the effects “failed to replicate” may be somewhat too strong, and a more cautious interpretation may be warranted. That said, the authors’ effort to test replicability across diverse methods is commendable, and the fact that these effects did not emerge consistently across such varied conditions provides important insights into the robustness of the original findings. Below I provide specific comments.

1. Clarification of methodological differences from the original studies: Although the present work attempts to replicate prior findings using multiple approaches, many of the studies do not closely match the original studies in terms of participants, platforms, and procedures. For example, Study 4 is described as a direct replication of Valdesolo & Graham (2014) Study 1; however, the original study recruited participants on a university campus and conducted the experiment on “iPads,” whereas the present Study 4 was conducted online using Prolific. It would therefore strengthen the manuscript to more clearly describe the participant characteristics, research environments, platforms, and measured variables in the original studies, and to explicitly specify how Studies 1–6 differ from them. This would support a more cautious and balanced interpretation of the findings.

2. Strength of the awe manipulation (manipulation check): It would be important to report how the effect sizes of the subjective awe measures compare with those reported in the original studies. Were they significantly different? If the awe induction was relatively weaker in the present research, this may help explain the pattern of results.

3. Additional analyses linking awe-related measures and outcomes: Relatedly, I encourage the authors to examine correlations between Awe, AWE-S, Small Self, and all outcome variables. It is possible that participants who experienced stronger awe may have shown effects more consistent with the original findings.

4. Tone of the conclusions: Given the methodological differences noted above and the potential value of additional analyses, the conclusion that the effects did not replicate may be too strong. After fully discussing these issues in the limitations section, it may be more appropriate to frame the findings as showing that the effects were “not replicated under the present conditions.”

Reviewer #2: The authors have presented a fascinating and much-needed piece of work, taking the topical theme of awe and examining its effects on a wide range of positive outcomes. At a time when it is commonplace to discuss high-level positive experiences such as well-being and prosociality, it is commendable that the researchers have sought to test the validity of these claims empirically. Overall, this is an important and promising piece of work that has the potential to make a valuable contribution to the field. However, there are a number of points on which I seek clarification, outlined below. I hope that addressing the comments could further strengthen the manuscript.

In the literature review, the authors cite influential works in the field, such as Keltner and Haidt. However, there are a number of recent works that could help further contextualise the present work. For example, considering that the authors characterise awe as a complex emotional experience that includes both fear and positivity, and that awe relates to the sublime, the authors may consider the following works that support that.

Gordon, A. M., Stellar, J. E., Anderson, C. L., McNeil, G. D., Loew, D., & Keltner, D. (2017). The dark side of the sublime: Distinguishing a threat-based variant of awe. Journal of Personality and Social Psychology, 113(2), 310–328. https://doi.org/10.1037/pspp0000120

Hur, Y.-J., Gerger, G., Leder, H., & McManus, I. C. (2020). Facing the sublime: Physiological correlates of the relationship between fear and the sublime. Psychology of Aesthetics, Creativity, and the Arts, 14(3), 253–263. https://doi.org/10.1037/aca0000204

It should be noted that a work that connects the sublime with awe is the Clewis et al. (2022) work, which the authors have included in the reference section, but which I cannot see in the main text.

Considering that the authors view awe as being triggered through nature-related vast objects, it may be worth considering the following empirical works, which find a link between objective/subjective vastness and awe/sublime:

Ishizu, T., & Zeki, S. (2014). A neurobiological enquiry into the origins of our experience of the sublime and beautiful. Frontiers in Human Neuroscience, 8, 891. https://doi.org/10.3389/fnhum.2014.00891

Hur, Y.-J., Hallam-Evans, C., Garfen, Y., Baiza, A., Backhouse Spriggs, T., Mircea, M.-T., Nagy, O., Pye, E., & McManus, I. C. (2024). Differentiating the visual aesthetics of the sublime and the beautiful: Selective effects of stimulus size, height, and color on sublimity and beauty ratings in photographs. Psychology of Aesthetics, Creativity, and the Arts, 18(6), 904–920. https://doi.org/10.1037/aca0000480

Overall, I found the citation system difficult to follow. The in-text citations appear to draw on two different systems; under APA style, citations should identify authors rather than use numeric references. Also, because some citations are presented numerically, it is not possible to reliably match the in-text citations to the reference list.

Regarding the studies, whether systematic or direct replication studies, I appreciate that the same emotional induction technique and measures were used (p. 9). Please clarify if the same analytical methods were also used, as the analytical methods can often be linked with different outcomes.

I have noticed that whereas all the studies presented were run online, it may not always be the case that the original studies were run online. If the study context has changed, this should be mentioned and discussed in the paper (especially in light of the lack of replication of results).

The paper by Piff et al. examines prosocial values across a large number of studies. Please clarify the rationale for selecting only Study 2 from Piff et al. for replication. Although the authors state (p. 28) that only studies amenable to online replication were selected, it appears that several of the other studies reported by Piff et al. could also have been conducted online. Could the authors clarify why Study 2 was selected in the Piff paper?

For all tables (and wherever inferential statistics are involved), please present effect sizes.

On page 28, the following sentence is not suitable as an introduction to the next set of studies, as it reveals the outcomes in advance: “Despite these changes, we continued to find the same pattern wherein awe was reported to be significantly induced, but no difference in outcome was statistically significant - except in one case where the awe manipulation failed (see Study 6)”

Certain sections of Study 6 (verbal prompts for experimental) would be more appropriate as supplementary material.

In studies of awe, experimental inductions often rely on a single stimulus, which may increase the potential for sample-specific variance. Could the authors please comment on this issue in the General Discussion section?

The authors note (p. 44) that the magnitude of awe induction itself might account for the lack of observed changes in its psychological impact (e.g., well-being, prosociality). If so, it would be helpful for the authors to compare the effect size of awe induction in the present study with that reported in the original studies. It is possible that the original studies produced a larger effect, which could explain the observable changes in well-being, prosociality, and related outcomes.

On pg. 43, the authors state that 31 out of 33 studies did not replicate the original studies. Please clarify how this sits with what was said in the abstract, that results were not replicated “except in one case” – some readers might find this confusing to understand.

Given that the authors have conducted 33 studies with similar designs and aims, they might consider performing a meta-analysis (e.g., a random-effects meta-analysis with a forest plot) of their findings. This would help consolidate evidence that, across studies, (1) awe induction occurs, but (2) its impact on positive outcomes is negligible. By assessing the overall effect of the manipulation across multiple studies, such an analysis could also provide a valuable interpretation of the single study in which a replication effect was indeed observed.

**********

what does this mean?). If published, this will include your full peer review and any attached files.

If you choose “no”, your identity will remain anonymous but your review may still be made public.

**Do you want your identity to be public for this peer review?**  For information about this choice, including consent withdrawal, please see our Privacy Policy

Reviewer #1: No

Reviewer #2: No

**********

[NOTE: If reviewer comments were submitted as an attachment file, they will be attached to this email and accessible via the submission site. Please log into your account, locate the manuscript record, and check for the action link "View Attachments". If this link does not appear, there are no attachment files.]

To ensure your figures meet our technical requirements, please review our figure guidelines: https://journals.plos.org/plosone/s/figures

You may also use PLOS’s free figure tool, NAAS, to help you prepare publication quality figures: https://journals.plos.org/plosone/s/figures#loc-tools-for-figure-preparation.

NAAS will assess whether your figures meet our technical requirements by comparing each figure against our figure specifications.

# Author comment: Author response to Decision Letter 1

**DOI:** 10.1371/journal.pone.0356728.r002

13 Apr 2026

Reviewer #1: This study examined the replicability of the cognitive and behavioral effects of awe—namely increased agency detection (Valdesolo & Graham, 2014), increased prosocial behavior (Piff et al., 2015), and enhanced well-being (Rudd et al., 2012)—using multiple awe-induction methods (videos, texts, and virtual reality) across both online and laboratory settings. Overall, the findings suggest that these effects were not clearly replicated under the conditions of the present research.

At the same time, the study does not fully qualify as a strict direct replication due to differences in participant pools and research platforms. Therefore, the tone of the claim that the effects “failed to replicate” may be somewhat too strong, and a more cautious interpretation may be warranted. That said, the authors’ effort to test replicability across diverse methods is commendable, and the fact that these effects did not emerge consistently across such varied conditions provides important insights into the robustness of the original findings. Below I provide specific comments.

1. Clarification of methodological differences from the original studies: Although the present work attempts to replicate prior findings using multiple approaches, many of the studies do not closely match the original studies in terms of participants, platforms, and procedures. For example, Study 4 is described as a direct replication of Valdesolo & Graham (2014) Study 1; however, the original study recruited participants on a university campus and conducted the experiment on “iPads,” whereas the present Study 4 was conducted online using Prolific. It would therefore strengthen the manuscript to more clearly describe the participant characteristics, research environments, platforms, and measured variables in the original studies, and to explicitly specify how Studies 1–6 differ from them. This would support a more cautious and balanced interpretation of the findings.

We agree that we could better clarify which studies were strict, direct replications and which were very similar but not exact. We have made several changes to the manuscript to clarify this point:

• First, we have added a Table which describes the original studies (pg 5) and which ones we attempted to replicate (shaded and in bold).

• Second, we have clarified our use of the phrases "direct" for exact replications and "systematic" for merely similar replications (pg 7).

• Finally, we have included a stronger rationale for delivering Study 4 using an online sample versus a campus sample. (pg. x) Each of these changes are provided below for ease of reference.

Table 1

Overview of Original Studies

Agency Detection - Valdesolo & Graham (2014)

Study (N) Awe Induction Method Outcomes

Study 1*

(N=120) Videos:

• awe (panoramic scenes of natural beauty such as mountains)

• amusement (comedic scenes from nature such as monkeys playing)

• neutral (an interview) Beliefs in supernatural control increased in the awe condition

Study 2

(N=81) Same videos as Study 1 Uncertainty intolerance mediated the effect of awe on belief in supernatural control

Study 3

(N= 120) Same videos as Study 1 Agency detection mediates the effect of the manipulation condition on awe

Study 4

(N=76) Neutral or Awe Videos from Study 1 Uncertainty intolerance mediated the relationship between awe and agency detection

Study 5

(N=112) Videos:

• awe - commercial with natural scenes)

• amusement - commercial with happy scenes) awe increased supernatural beliefs

Prosociality - Piff et al. (2015)

Study (N) Awe Induction Method Outcomes

Study 1 N=1519 (no induction) Dispositional awe is associated with dispositional prosocial tendencies

Study 2*

(N=75) Writing Recall Task:

• awe - memory of an awe experience

• Pride - memory of a pride experience

• Neutral - memory of a recent event of any kind Ethical decision making increased only in the awe condition

Study 3

(N=264) Videos:

• Same as Valdesolo & Graham's (2014) awe and amusement videos

• Neutral - wooden countertop Increased prosocial values for the awe condition

Study 4 Videos

• awe - scientific footage of water droplets colliding at high magnification in slow motion, threatening natural scenes (e.g., tornado)

• neutral - wooden countertop Greater generosity in the dictator game for the awe condition

Study 5 In Vivo

• awe - look at tall trees

• neutral - look at campus building Increased helping behavior (picking up pens) and reduced entitlement in the awe condition

Life Satisfaction - Rudd et al., (2012)

Study (N) Awe Induction Method Outcomes

Study 1 (N=63) Videos

• awe - commercial with natural scenes

• control - commercial with happy scenes More perceived time availability in the awe condition

Study 2

(N=86) Writing Recall Task

• awe - memory of an awe experience

• control - memory of a happy experience Those in awe condition were more willing to donate time and money

Study 3*

(N=105) Story Visualization

• awe - read a story about climbing to the top of the Eiffel tower to look out at Paris

• control - read a story about an unexceptional tower and landscape Those in the awe condition reported more momentary life satisfaction, as well as preference for experiential vs. material goods

Note: Bolded and highlighted denote the specific original studies that systematic and direct replications were based on.

"In a series of 6 distinct studies, we attempt to replicate these outcomes associated with awe using two types of replications conceptual vs. direct (33). We use the term systematic replication to mean repeating studies conceptually, with intentional changes to the methodology to see whether the effects hold under new conditions. On the other hand, a direct replication involves repeating studies conceptually and methodologically as closely as possible to see if the same result can be reproduced. In the first series of studies in this paper, we conduct systematic replications, which were designed to expand upon the work of previous authors by including novel hypotheses and multiple outcomes (see Table 1). In the case of direct replications, we consulted with and received materials from the original lead authors to conduct high-fidelity versions of their original work. For all replications the same analytic techniques were used to examine differences between three (ANOVAs) or two (t-tests) between conditions, as well as the same partial eta squared (ηp²) effect size measure to allow for comparison across studies." (PG 7)

"Study 4 in our study was a direct replication of Valdesolo and Graham's (2014; Study 1) video manipulation to elicit awe, amusement, or no emotion (neutral). Several authors (M.G., D.B.Y.) have already conducted an exact direct replication using a college campus sample as the authors did in the original study, similarly finding that the awe manipulation was successful, but outcomes did not replicate (Yaden et al., in press). To replicate this study with a bigger sample, an online sample was chosen with the acknowledgment that though this is a slight methodological change, in the original study the students on campus were still given an iPad to view the videos online. Given that this was the only change, we classify and report this study as a direct replication as the methodology was followed as closely as possible (see definition on page 7).

Like the original study, we expected the experimental awe condition would result in changes to supernatural control and belief in God relative to the active and passive control conditions." (PG 32)

2. Strength of the awe manipulation (manipulation check): It would be important to report how the effect sizes of the subjective awe measures compare with those reported in the original studies. Were they significantly different? If the awe induction was relatively weaker in the present research, this may help explain the pattern of results.

Thank you for this helpful suggestion. We have now created a Table to provide a reader friendly comparison of the effect sizes from the original studies that we replicate, and those studies reported int he paper on page 3. The table is reproduced below:

Table 3

Awe Manipulation Effect Size Comparison to Original Studies

Original Study Replication

Valdesolo & Graham (2014)

• Study 1 - ηp²=.47 (large) Systematic

• Study 1 - ηp²=.25 (large)

Direct

• Study 4 - ηp²=.30 (large)

Piff et al. (2014)

• Study 2 - NR Systematic

• Study 2 - ηp²=.15 (large)

Direct

• Study 5 - ηp²=.31 (large)

Rudd et al. (2012)

• Study 3 - ηp²=.04 (small) Systematic

• Study 3 - ηp²= .09 (medium)

Direct

• Study 6 - ηp²=.01NS (small)

Note. NR = Not reported. NS = Nonsignificant effect. Effect magnitude labels correspond to conventional benchmarks for partial eta squared (ηp²): small = .01, medium = .06, large = .14 (37,38),

3. Additional analyses linking awe-related measures and outcomes: Relatedly, I encourage the authors to examine correlations between Awe, AWE-S, Small Self, and all outcome variables. It is possible that participants who experienced stronger awe may have shown effects more consistent with the original findings.

As this is a series of replications, we did not want to break with the original studies which do not conduct such analyses. However, given that the small-self has been identified as a feature or facet of awe in the time since these studies were published, we do agree that examining the correlation between small-self and outcomes is warranted. We now provide a correlation matrix in the supplement, and have produced it here below:

Table 3

Correlations between Awe Item, AWE-S, Small Self and Primary Outcome Variables

Study 1 - Outcomes Awe Item Awe-S Small Self

Life Satisfaction .188** .163** .089*

Supernatural Control .289** .376** .254**

Belief in God .265** .347** .240**

Supernatural Belief .313** .403** .279**

Prosocial Behavior .187** .254** .130**

Intentional Pattern Perception .114** .154** .134**

Uncertainty Intolerance .040 .042 .030

Mind Perception .186** .301** .242**

Study 2 - Outcomes

Life Satisfaction .213** .259** .203**

Supernatural Control .195** .391** .293**

Supernatural Belief .227** .419** .327**

Prosocial Behavior .171** .255** .169**

Intentional Pattern Perception .199** .274** .227**

Uncertainty Intolerance .047 .192** .188**

Mind Perception .269** .416** .380**

Study 3 - Outcomes

Life Satisfaction .038 .336** .319**

Supernatural Control .244* .105 .250*

Moral Circle .003 .112 .205

Primals - Interconnected .044 .132 .259*

Primals - Safe -.022 .142 -.081

Primals - Alive .045 .153 .185

Intentionality .088 .207* .333*

Presence .250* .214* .327*

Inclusion of Self In Other -.195 -.087 -.086

Study 4 - Outcomes

Supernatural Control .178** - -

Belief in God .139* - -

Study 5 - Outcomes

Ethical Decision Making .071 - .008

Study 6 - Outcomes

Momentary Life Satisfaction .011 - -

Perceived Time Availability .078 - -

Preference for Experiential Goods .038 - -

p<.05*; p<.01**; - = not measured

4. Tone of the conclusions: Given the methodological differences noted above and the potential value of additional analyses, the conclusion that the effects did not replicate may be too strong. After fully discussing these issues in the limitations section, it may be more appropriate to frame the findings as showing that the effects were “not replicated under the present conditions.”

We have now elaborated on the difference between systematic and direct replications (see Comment 1) and have accordingly reframed our discussion of the findings considering the reviewer’s comments. For systematic (non-direct) replications we have added language to indicate that the effects were not replicated under the present conditions. As an example:

"Under the present conditions, we did not replicate the finding reported in Valdesolo and Graham (2014), Piff and colleagues (2015); and Rudd and colleagues (2012), though it should be noted that they used a few different forms of emotion inductions, especially video but also writing exercises." (PG 17)

Reviewer #2: The authors have presented a fascinating and much-needed piece of work, taking the topical theme of awe and examining its effects on a wide range of positive outcomes. At a time when it is commonplace to discuss high-level positive experiences such as well-being and prosociality, it is commendable that the researchers have sought to test the validity of these claims empirically. Overall, this is an important and promising piece of work that has the potential to make a valuable contribution to the field. However, there are a number of points on which I seek clarification, outlined below. I hope that addressing the comments could further strengthen the manuscript.

In the literature review, the authors cite influential works in the field, such as Keltner and Haidt. However, there are a number of recent works that could help further contextualise the present work. For example, considering that the authors characterise awe as a complex emotional experience that includes both fear and positivity, and that awe relates to the sublime, the authors may consider the following works that support that.

Gordon, A. M., Stellar, J. E., Anderson, C. L., McNeil, G. D., Loew, D., & Keltner, D. (2017). The dark side of the sublime: Distinguishing a threat-based variant of awe. Journal of Personality and Social Psychology, 113(2), 310–328. https://doi.org/10.1037/pspp0000120

Hur, Y.-J., Gerger, G., Leder, H., & McManus, I. C. (2020). Facing the sublime: Physiological correlates of the relationship between fear and the sublime. Psychology of Aesthetics, Creativity, and the Arts, 14(3), 253–263. https://doi.org/10.1037/aca0000204

It should be noted that a work that connects the sublime with awe is the Clewis et al. (2022) work, which the authors have included in the reference section, but which I cannot see in the main text.

Considering that the authors view awe as being triggered through nature-related vast objects, it may be worth considering the following empirical works, which find a link between objective/subjective vastness and awe/sublime:

Ishizu, T., & Zeki, S. (2014). A neurobiological enquiry into the origins of our experience of the sublime and beautiful. Frontiers in Human Neuroscience, 8, 891. https://doi.org/10.3389/fnhum.2014.00891

Hur, Y.-J., Hallam-Evans, C., Garfen, Y., Baiza, A., Backhouse Spriggs, T., Mircea, M.-T., Nagy, O., Pye, E., & McManus, I. C. (2024). Differentiating the visual aesthetics of the sublime and the beautiful: Selective effects of stimulus size, height, and color on sublimity and beauty ratings in photographs. Psychology of Aesthetics, Creativity, and the Arts, 18(6), 904–920. https://doi.org/10.1037/aca0000480

We have added content to the introduction which includes the citations you have suggested:

"The notion of awe can be traced back to the concept of “the sublime” in aesthetics, as articulated by Edmund Burke, Immanuel Kant, and others (3). Contemporary scholarship has continued to examine the link between awe and the sublime, suggesting that these experiences share core features including perceptual vastness, cognitive accommodation, and complex states of simultaneous positive and negative emotion, including elements of fear (3–5). Empirical work further supports the importance of subjective and objective features (e.g., height, scale, magnitude) in creating a sense of vastness in both sublime and awe experiences (6,7). Awe as a distinct psychological construct was first systematically outlined in the seminal paper “Approaching awe, a moral, spiritual, and aesthetic emotion” by Keltner and Haidt. They articulate two necessary qualities of awe: (1) perceptual vastness and (2) a need for accommodation. First, awe involves contact with a perceptually vast stimulus and second, a need for accommodation on behalf of the experiencer, which entails the need for a cognitive shift in one’s understanding of these stimuli (2)." (Pgs. 3-4).

Overall, I found the citation system difficult to follow. The in-text citations appear to draw on two different systems; under APA style, citations should identify aut

Attachment Submitted filename: Response to Reviewers.docx

# Peer review: Decision Letter 1

**DOI:** 10.1371/journal.pone.0356728.r003

**Authors:** Haixia Wang

16 Jun 2026

Dear Dr. Graziosi,

Thank you for submitting your manuscript to PLOS ONE. After careful consideration, we feel that it has merit but does not fully meet PLOS ONE’s publication criteria as it currently stands. Therefore, we invite you to submit a revised version of the manuscript that addresses the points raised during the review process.

==============================

Based on the reviewers’ assessments and my own evaluation, I am pleased to invite you to revise your manuscript major revision. The reviewers have raised several constructive points that need to be addressed before the paper can be considered for publication.

In particular, please note that PLOS ONE considers psychology or behavioural research studies conducted using Amazon MTurk / Survey Monkey for publication. However, during the evaluation of your manuscript, we would be grateful if you could carefully address the limitations commonly associated with this type of study—namely, the non‑naivety and trustworthiness of participants. You should explicitly discuss how these issues were handled in your study design, data collection, and quality assurance procedures. Additionally, please consider whether you need to validate the results using another non‑web‑based sample (e.g., a laboratory or community sample). If you believe such validation is not necessary, please provide a clear and compelling justification in your revision.

==============================

Please submit your revised manuscript by Jul 31 2026 11:59PM. If you will need more time than this to complete your revisions, please reply to this message or contact the journal office at plosone@plos.org. When you're ready to submit your revision, log on to https://www.editorialmanager.com/pone/ and select the 'Submissions Needing Revision' folder to locate your manuscript file.

- A letter that responds to each point raised by the academic editor and reviewer(s). You should upload this letter as a separate file labeled 'Response to Reviewers'.
- A marked-up copy of your manuscript that highlights changes made to the original version. You should upload this as a separate file labeled 'Revised Manuscript with Track Changes'.
- An unmarked version of your revised paper without tracked changes. You should upload this as a separate file labeled 'Manuscript'.

If you would like to make changes to your financial disclosure, please include your updated statement in your cover letter. Guidelines for resubmitting your figure files are available below the reviewer comments at the end of this letter.

If applicable, we recommend that you deposit your laboratory protocols in protocols.io to enhance the reproducibility of your results. Protocols.io assigns your protocol its own identifier (DOI) so that it can be cited independently in the future. For instructions see: https://journals.plos.org/plosone/s/submission-guidelines#loc-laboratory-protocols. Additionally, PLOS ONE offers an option for publishing peer-reviewed Lab Protocol articles, which describe protocols hosted on protocols.io. Read more information on sharing protocols at https://plos.org/protocols?utm_medium=editorial-email&utm_source=authorletters&utm_campaign=protocols.

As the corresponding author, your ORCID iD is verified in the submission system and will appear in the published article. PLOS supports the use of ORCID, and we encourage all coauthors to register for an ORCID iD and use it as well. Please encourage your coauthors to verify their ORCID iD within the submission system before final acceptance, as unverified ORCID iDs will not appear in the published article. *Only*  the individual author can complete the verification step; PLOS staff *cannot*  verify ORCID iDs on behalf of authors.

We look forward to receiving your revised manuscript.

Kind regards,

Haixia Wang

Academic Editor

PLOS One

Journal Requirements:

If the reviewer comments include a recommendation to cite specific previously published works, please review and evaluate these publications to determine whether they are relevant and should be cited. There is no requirement to cite these works unless the editor has indicated otherwise.

Additional Editor Comments (if provided):

[Note: HTML markup is below. Please do not edit.]

Reviewers' comments:

Reviewer's Responses to Questions

**Comments to the Author**

Reviewer #1: All comments have been addressed

Reviewer #2: (No Response)

**********

2. Is the manuscript technically sound, and do the data support the conclusions?

Reviewer #1: Yes

Reviewer #2: Yes

**********

3. Has the statistical analysis been performed appropriately and rigorously?

Reviewer #1: Yes

Reviewer #2: Yes

**********

4. Have the authors made all data underlying the findings in their manuscript fully available?

The PLOS Data policy

Reviewer #1: Yes

Reviewer #2: Yes

**********

5. Is the manuscript presented in an intelligible fashion and written in standard English?

Reviewer #1: Yes

Reviewer #2: Yes

**********

Reviewer #1: Thank you for the careful revisions. The manuscript has improved substantially, and my main concerns have largely been addressed. I have only a few remaining minor comments.

1. In the response letter, the authors state that they have clarified their use of the terms “direct” for exact replications and “systematic” for merely similar replications. However, this clarification does not appear to be fully reflected in the manuscript. In the manuscript, the distinction is still framed primarily as “conceptual” versus “direct” replication, and the relationship between “conceptual” and “systematic” replication remains unclear. Please ensure that these terms are used consistently and that the intended distinction is clearly reflected in the manuscript.

2. For Study 4, if it is retained as a direct replication, please more explicitly acknowledge the methodological difference from the original study, namely the shift from a campus/iPad setting to an online Prolific sample. This difference should also be briefly noted in the limitations, and statements such as “this was the only change” may need to be phrased more cautiously.

3. Please correct the inconsistent citation year for Piff et al., which appears as both 2014 and 2015. The manuscript should use the correct year consistently.

4. The newly added exploratory correlations between self-reported awe, AWE-S, small self, and the outcome variables are informative. Although these analyses are correlational and exploratory, they suggest that, at the individual-difference level, participants who experienced stronger awe may have shown higher scores on some relevant outcomes. I recommend briefly mentioning these findings in the main text and discussing their interpretation cautiously in the Discussion, rather than leaving them only in the supplement.

Overall, I find the authors’ revisions responsive, and these remaining issues appear minor but important for clarity and balanced interpretation.

Reviewer #2: The authors have addressed the initial concerns, and I thank them for their corrections. Please see my minor comments below:

- On page 4, the authors ought to delete the “4/13/2026 1:33:00 PM”, as I am almost certain this is a typing mistake. Full section as it currently stands: “Other facets of awe include alterations to time (e.g., the sense that time slows down or speeds up; 4/13/2026 1:33:00 PM, small self…”.

- The authors have ruled out the possibility of a meta-analysis in the present analysis, citing that it is outside of the scope of the present manuscript. Could the authors please further elaborate?

**********

what does this mean?). If published, this will include your full peer review and any attached files.

If you choose “no”, your identity will remain anonymous but your review may still be made public.

**Do you want your identity to be public for this peer review?**  For information about this choice, including consent withdrawal, please see our Privacy Policy

Reviewer #1: No

Reviewer #2: No

**********

[NOTE: If reviewer comments were submitted as an attachment file, they will be attached to this email and accessible via the submission site. Please log into your account, locate the manuscript record, and check for the action link "View Attachments". If this link does not appear, there are no attachment files.]

To ensure your figures meet our technical requirements, please review our figure guidelines: https://journals.plos.org/plosone/s/figures

You may also use PLOS’s free figure tool, NAAS, to help you prepare publication quality figures: https://journals.plos.org/plosone/s/figures#loc-tools-for-figure-preparation.

NAAS will assess whether your figures meet our technical requirements by comparing each figure against our figure specifications.

# Author comment: Author response to Decision Letter 2

**DOI:** 10.1371/journal.pone.0356728.r004

26 Jun 2026

Editor Comments:

In particular, please note that PLOS ONE considers psychology or behavioural research studies conducted using Amazon MTurk / Survey Monkey for publication. However, during the evaluation of your manuscript, we would be grateful if you could carefully address the limitations commonly associated with this type of study—namely, the non naivety and trustworthiness of participants. You should explicitly discuss how these issues were handled in your study design, data collection, and quality assurance procedures.

Thank you, we have added language (reproduced below) to address the limitations of MTurk in the two studies in which we recruited participants via MTurk (Study 1 and Study 2). Note that the other online samples were collected via Prolific (Study 4-6) and Study 3 was an in-person laboratory study.

"Although data for two of the six studies were collected via Amazon Mechanical Turk, concerns regarding participant non-naivety and response trustworthiness were addressed through multiple quality assurance procedures (e.g., attention checks, eligibility screening, and exclusion of low-quality responses), although these concerns remain an inherent limitation of online convenience samples." (Pg. 9)

Additionally, please consider whether you need to validate the results using another non web based sample (e.g., a laboratory or community sample). If you believe such validation is not necessary, please provide a clear and compelling justification in your revision.

As above, note that we did conduct a laboratory study reported in this manuscript. We have also conducted an in-person laboratory study cited in the manuscript with the same results.

We do not believe that further validation using a non-web-based sample is necessary in this case. First, the pattern of results was highly consistent across methodologies and settings. Specifically, null findings were observed not only in large online samples but also in a laboratory-based virtual reality study (Study 3) and are consistent with independent in-person replication attempt we have published elsewhere. Taken together, these convergent findings suggest that the lack of replication is unlikely to reflect artifacts of online data collection. We include reference to the results of our in-person pre-registered direct replication of Valdesolo and Graham's Study. The language is reproduced below:

"Study 4 in our study was a designed as a direct replication of Valdesolo and Graham's (2014; Study 1) video manipulation to elicit awe, amusement, or no emotion (neutral). Several authors (M.G., D.B.Y., along an original author, Valdesolo) have previously conducted a pre-registered direct replication using an in-person college campus sample as the authors did in the original study, similarly finding that the awe manipulation was successful, but outcomes from the original study did not replicate (Yaden et al., in press). To increase statistical power and sample size, an online Prolific sample was chosen rather than recruiting participants from a college campus. We acknowledge that this represents a methodological deviation from the original study. However, the participants in our study viewed the exact same video stimuli that the original study participants viewed on iPads, and the measures, procedures, and analytic approach were otherwise matched to those used by the original authors. Consistent with our definition of direct replication (p.7), we classify this study as a direct replication while recognizing that the change may have influenced the findings." (Pg. 29)

Reviewer #1

Thank you for the careful revisions. The manuscript has improved substantially, and my main concerns have largely been addressed. I have only a few remaining minor comments.

1. In the response letter, the authors state that they have clarified their use of the terms “direct” for exact replications and “systematic” for merely similar replications. However, this clarification does not appear to be fully reflected in the manuscript. In the manuscript, the distinction is still framed primarily as “conceptual” versus “direct” replication, and the relationship between “conceptual” and “systematic” replication remains unclear. Please ensure that these terms are used consistently and that the intended distinction is clearly reflected in the manuscript.

Thank you for highlighting this inconsistency. In the response letter the use of the word systematic was an error. We have eliminated the word "systematic" throughout the manuscript and replaced it with "conceptual", which is terminology grounded in the research literature to describe these different approaches to replication. We have clarified this further in the body of the manuscript and included additional citations for this language.

"In a series of 6 distinct studies, we attempt to replicate these outcomes associated with awe using two types of replications: conceptual vs. direct (33, 34, 35). The term "conceptual replication" is used throughout the literature to mean repeating studies conceptually, with intentional changes to the methodology to see whether the effects hold under new conditions. On the other hand, a direct replication involves repeating studies conceptually and methodologically as closely as is possible to see if the same result can be reproduced. In the first series of studies in this paper, we conduct conceptual replications, which were designed to expand upon the work of previous authors by including novel hypotheses and multiple outcomes (see Table 1). In the case of direct replications, we consulted with and received materials from the original lead authors to conduct high-fidelity versions of their original work as closely as possible. For all replications the same analytic techniques were used to examine differences between three (ANOVAs) or two (t-tests) conditions, as well as the same partial eta squared (ηp²) effect size measure to allow for comparison across studies." (P. 7)

2. For Study 4, if it is retained as a direct replication, please more explicitly acknowledge the methodological difference from the original study, namely the shift from a campus/iPad setting to an online Prolific sample. This difference should also be briefly noted in the limitations, and statements such as “this was the only change” may need to be phrased more cautiously.

"Study 4 in our study was a designed as a direct replication of Valdesolo and Graham's (2014; Study 1) video manipulation to elicit awe, amusement, or no emotion (neutral). Several authors (M.G., D.B.Y., along an original author, Valdesolo) have previously conducted a pre-registered direct replication using an in-person college campus sample as the authors did in the original study, similarly finding that the awe manipulation was successful, but outcomes from the original study did not replicate (Yaden et al., in press). To increase statistical power and sample size, an online Prolific sample was chosen rather than recruiting participants from a college campus. We acknowledge that this represents a methodological deviation from the original study. However, the participants in our study viewed the exact same video stimuli that the original study participants viewed on iPads, and the measures, procedures, and analytic approach were otherwise matched to those used by the original authors. Consistent with our definition of direct replication (p.7), we classify this study as a direct replication while recognizing that the change may have influenced the findings." (P. 29)

3. Please correct the inconsistent citation year for Piff et al., which appears as both 2014 and 2015. The manuscript should use the correct year consistently.

Thank you, this has been updated throughout.

4. The newly added exploratory correlations between self-reported awe, AWE-S, small self, and the outcome variables are informative. Although these analyses are correlational and exploratory, they suggest that, at the individual-difference level, participants who experienced stronger awe may have shown higher scores on some relevant outcomes. I recommend briefly mentioning these findings in the main text and discussing their interpretation cautiously in the Discussion, rather than leaving them only in the supplement.

Thank you for raising this point. We have included language in the results and discussion section referencing the correlational analyses (reproduced below):

"Although the experimental manipulations generally did not produce significant between-group differences, exploratory analyses indicated that participants reporting greater subjective experiences of awe tended to report higher scores on several outcome measures (see Supplementary Table 3 for exploratory correlations for each of the six studies in this paper)." (P. 15)

"Although experimental manipulations largely failed to produce differences between conditions, exploratory correlations indicated that individuals who reported stronger experiences of awe tended to score higher on several outcomes. Though causal conclusions cannot be drawn, these analyses raise the possibility that the magnitude or intensity of the awe experience may be an important feature to examine in future research." (P. 43)

Overall, I find the authors’ revisions responsive, and these remaining issues appear minor but important for clarity and balanced interpretation.

Reviewer #2

The authors have addressed the initial concerns, and I thank them for their corrections. Please see my minor comments below:

On page 4, the authors ought to delete the “4/13/2026 1:33:00 PM”, as I am almost certain this is a typing mistake. Full section as it currently stands: “Other facets of awe include alterations to time (e.g., the sense that time slows down or speeds up; 4/13/2026 1:33:00 PM, small self…"

Thank you for catching this artifact of our reference manager use. This sentence has been corrected.

The authors have ruled out the possibility of a meta-analysis in the present analysis, citing that it is outside of the scope of the present manuscript. Could the authors please further elaborate?

We appreciate the suggestion and agree that a meta-analysis of the broader field would be valuable. However, in the context of this manuscript we do not believe that a meta-analysis of the six studies would be appropriate because the studies have different outcomes. These outcomes include the following: well-being, supernatural control, belief in God, prosociality, and small self. The studies include different outcomes and even different outcome measures similar concepts preventing a meaningful meta-analysis.

Attachment Submitted filename: Response to Reviewers (2).docx

# Peer review: Decision Letter 2

**DOI:** 10.1371/journal.pone.0356728.r005

**Authors:** Haixia Wang

6 Aug 2026

A Series of Replication Attempts of Experimental Awe-Induction Effects

PONE-D-25-65368R2

Dear Dr. Graziosi,

We’re pleased to inform you that your manuscript has been judged scientifically suitable for publication and will be formally accepted for publication once it meets all outstanding technical requirements.

Within one week, you’ll receive an e-mail detailing the required amendments. When these have been addressed, you’ll receive a formal acceptance letter and your manuscript will be scheduled for publication.

An invoice will be generated when your article is formally accepted. Please note, if your institution has a publishing partnership with PLOS and your article meets the relevant criteria, all or part of your publication costs will be covered. Please make sure your user information is up-to-date by logging into Editorial Manager at Editorial Manager® and clicking the ‘Update My Information' link at the top of the page. For questions related to billing, please contact billing support.

If your institution or institutions have a press office, please notify them about your upcoming paper to help maximize its impact. If they’ll be preparing press materials, please inform our press team as soon as possible -- no later than 48 hours after receiving the formal acceptance. Your manuscript will remain under strict press embargo until 2 pm Eastern Time on the date of publication. For more information, please contact onepress@plos.org.

Kind regards,

Haixia Wang

Academic Editor

PLOS One

Additional Editor Comments (optional):

Reviewers' comments:

Reviewer's Responses to Questions

**Comments to the Author**

Reviewer #1: All comments have been addressed

Reviewer #2: All comments have been addressed

**********

2. Is the manuscript technically sound, and do the data support the conclusions?

Reviewer #1: Yes

Reviewer #2: Yes

**********

3. Has the statistical analysis been performed appropriately and rigorously?

Reviewer #1: Yes

Reviewer #2: Yes

**********

4. Have the authors made all data underlying the findings in their manuscript fully available?

The PLOS Data policy

Reviewer #1: Yes

Reviewer #2: Yes

**********

5. Is the manuscript presented in an intelligible fashion and written in standard English?

Reviewer #1: Yes

Reviewer #2: Yes

**********

Reviewer #1: (No Response)

Reviewer #2: (No Response)

**********

what does this mean?). If published, this will include your full peer review and any attached files.

If you choose “no”, your identity will remain anonymous but your review may still be made public.

**Do you want your identity to be public for this peer review?**  For information about this choice, including consent withdrawal, please see our Privacy Policy

Reviewer #1: No

Reviewer #2: No

**********

# Editor report: Acceptance letter

**DOI:** 10.1371/journal.pone.0356728.r006

**Authors:** Haixia Wang

PONE-D-25-65368R2

PLOS One

Dear Dr. Graziosi,

I'm pleased to inform you that your manuscript has been deemed suitable for publication in PLOS One. Congratulations! Your manuscript is now being handed over to our production team.

At this stage, our production department will prepare your paper for publication. This includes ensuring the following:

* All references, tables, and figures are properly cited

* All relevant supporting information is included in the manuscript submission,

* There are no issues that prevent the paper from being properly typeset

You will receive further instructions from the production team, including instructions on how to review your proof when it is ready. Please keep in mind that we are working through a large volume of accepted articles, so please give us a few days to review your paper and let you know the next and final steps.

Lastly, if your institution or institutions have a press office, please let them know about your upcoming paper now to help maximize its impact. If they'll be preparing press materials, please inform our press team within the next 48 hours. Your manuscript will remain under strict press embargo until 2 pm Eastern Time on the date of publication. For more information, please contact onepress@plos.org.

You will receive an invoice from PLOS for your publication fee after your manuscript has reached the completed accept phase. If you receive an email requesting payment before acceptance or for any other service, this may be a phishing scheme. Learn how to identify phishing emails and protect your accounts at https://explore.plos.org/phishing.

If we can help with anything else, please email us at customercare@plos.org.

Thank you for submitting your work to PLOS One and supporting open access.

Kind regards,

PLOS ONE Editorial Office Staff

on behalf of

Dr. Haixia Wang

Academic Editor

PLOS One
